# Length Variations amongst Protein Domain Superfamilies and Consequences on Structure and Function

**DOI:** 10.1371/journal.pone.0004981

**Published:** 2009-03-31

**Authors:** Sankaran Sandhya, Saane Sudha Rani, Barah Pankaj, Madabosse Kande Govind, Bernard Offmann, Narayanaswamy Srinivasan, Ramanathan Sowdhamini

**Affiliations:** 1 National Centre for Biological Sciences (TIFR), GKVK Campus, Bangalore, India; 2 Laboratoire de Biochimie et Génétique Moléculaire BP 7151, Université de La Réunion, La Réunion, France; 3 Molecular Biophysics Unit, Indian Institute of Science, Bangalore, India; Griffith University, Australia

## Abstract

**Background:**

Related protein domains of a superfamily can be specified by proteins of diverse lengths. The structural and functional implications of indels in a domain scaffold have been examined.

**Methodology:**

In this study, domain superfamilies with large length variations (more than 30% difference from average domain size, referred as ‘length-deviant’ superfamilies and ‘length-rigid’ domain superfamilies (<10% length difference from average domain size) were analyzed for the functional impact of such structural differences. Our delineated dataset, derived from an objective algorithm, enables us to address indel roles in the presence of peculiar structural repeats, functional variation, protein-protein interactions and to examine ‘domain contexts’ of proteins tolerant to large length variations. Amongst the top-10 length-deviant superfamilies analyzed, we found that 80% of length-deviant superfamilies possess distant internal structural repeats and nearly half of them acquired diverse biological functions. In general, length-deviant superfamilies have higher chance, than length-rigid superfamilies, to be engaged in internal structural repeats. We also found that ∼40% of length-deviant domains exist as multi-domain proteins involving interactions with domains from the same or other superfamilies. Indels, in diverse domain superfamilies, were found to participate in the accretion of structural and functional features amongst related domains. With specific examples, we discuss how indels are involved directly or indirectly in the generation of oligomerization interfaces, introduction of substrate specificity, regulation of protein function and stability.

**Conclusions:**

Our data suggests a multitude of roles for indels that are specialized for domain members of different domain superfamilies. These specialist roles that we observe and trends in the extent of length variation could influence decision making in modeling of new superfamily members. Likewise, the observed limits of length variation, specific for each domain superfamily would be particularly relevant in the choice of alignment length search filters commonly applied in protein sequence analysis.

## Introduction

During evolution, protein domains undergo many modifications in sequence and structure to achieve versatility in function. Diverse factors, such as the accumulation of sequence changes, gene duplications, gene combinations *etc.*, are seen to contribute extensively to this diversity [1]–[6]. Intriguingly, examination of the wealth of structures deposited in the PDB [7] shows that the increasing pace of protein structure determination is not necessarily associated with an increase in the number of novel folds. Although, estimates for the number of protein folds vary [6], [8], it is unlikely that this number will supersede sequence space. Hierarchical assemblies of protein structures in databanks such as SCOP [9] and CATH [10] only emphasize the diversity of proteins sharing similar structures and the tolerance of stable folds to variation not only in sequence but also in domain lengths. Therefore, functional versatility is attributed to novel interfaces resulting from domain recombination and the mixing and modulation of pre-existing scaffolds through length modifications.

Length differences between domains are introduced through insertions and deletions (indels) into pre-existing domains. It has been shown that protein length expansions are 40–60% greater in eukaryotes than in prokaryotes and that such expansions correlate with the presence of introns and accretion of functional motifs that are involved in sophisticated regulatory networks [11]. Recent studies have also shown that protein structural differences can emerge through an incremental growth of protein variable regions. In phylogenetic reconstructions of SCOP domain families, 42% of observed insertions occur in insert regions and contribute to structural innovations [12].

In an analysis of length differences in 353 multi-membered PASS2 domain superfamily alignments [13], we had observed that such domain length differences or ‘indels’ occur in all protein classes. Indeed, ∼60% of protein domains from all protein classes showed at least 5% length variations from their typical domain size. The extent of length variation varied from two–three residues to over two-fold. Also, in this study, it was seen that some domains are flexible and tolerant to length variation (‘length-deviant’ domains) while others are less permissive to length changes (‘length-rigid’ domains). There also appeared to be a correlation between protein class and the nature/ preferred structural type in indels that can aid in decision making in modeling for the choice of structures in indel regions. Indeed, indels in α-helical proteins were preferentially coils (∼60%) and classes with mixed topologies such as α/β and α+β prefer helices and coils in indel regions (>50%). Manual examination of alignments showed that such indels occur not only as extensions to pre-existing structures, but are introduced in existing domains into the middle of the structure. The strict maintenance of the core scaffold, despite permitting large indels, suggests that indels are likely to influence the structural/functional features of the domains in which they occur. Our statistical evaluation of indel properties, also showed that 60% of indels were of short length (<5 residues) suggesting that in most domains they are inserted as short, *albeit*, discontinuous insertions [13].

Length variation in proteins has been the object of several analyses and many groups have performed independent studies on domain and protein length variations. Pascarella and Argos [14] had also observed that ∼90% of indels in proteins of sequence identity ranging from 0–20% and 40–80% were of short length (<10 residues). Their study also showed that loops, coils and turns are evenly targeted for insertions and deletions. Reeves and co-workers [15], in a comprehensive examination of structural diversity in CATH domain superfamilies, have reported that a two-fold or more variation in the number of secondary structures was observed in 56% of well-populated superfamilies. Even though such insertions are discontinuous in sequence, they co-locate in three-dimensional (3-D) space to perform functional roles or generate novel interaction interfaces. Indels have also been implicated in directly influencing functional differences between homologous domains [16].

Here, we assess the functional and structural advantages of length variations amongst homologous members of 64 length-deviant domain superfamilies. The role of indels in mediating novel interaction interfaces through the formation of structural repeats, multi-domain combinations and higher order oligomers has been examined. The presence of distant internal repeats in length-deviant superfamilies has been carried out using computer algorithms, both using sequence and structural information. In addition to a manual comparison of the giant and dwarf representative domains in each length-rigid and length-deviant domain superfamily, literature has been consulted, where relevant, to support the structural observations and inferences on functional impact made here. Likewise, SCOP domain definitions and domain assignments have been consulted to understand the social contexts of domains. Further, the analysis has been extended to protein-protein interaction databases to examine if length-deviant domains are indeed social in functional contexts and associate with a high number of interacting partners. We have also reasoned whether additional lengths assist domains to interact with multiple copies of domains-either homologous or other. This would address if the ability to accommodate extra length reflects on the ‘social’ skills of a domain to interact with more neighboring domains.

## Results

**Figure 1:**
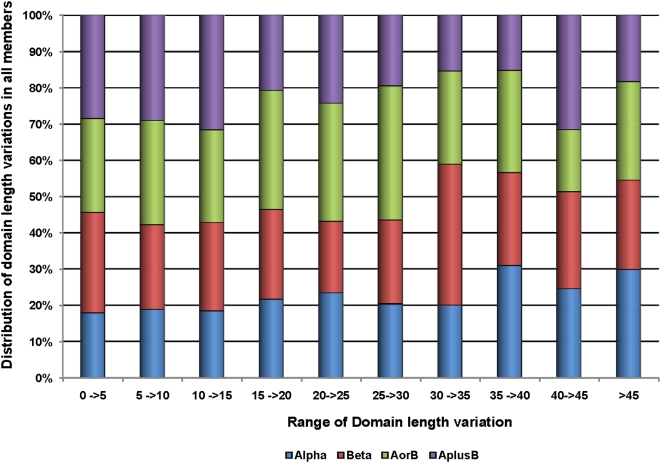
Distributions of domain length variations in members of the 353 multi-membered PASS2 domain superfamilies. The degree of length variation for every member from the mean domain size of its superfamily was calculated by expressing as a ratio the length difference of each member to its mean domain size.

We have investigated the functional and structural implications of indels amongst related members of a domain superfamily. We applied the CUSP algorithm to identify indels in domain members of 353 multi-membered domain superfamilies [13]. Structure-based sequence alignments for such superfamilies that are represented by more than one domain member are already available in the PASS2 database, where domains sharing <40% sequence identity have been aligned. Further, a quantitative description of the extent of length variation in each of the multi-membered protein domain superfamilies, to analyze the nature and typical lengths of indels in the four major structural classes, showed that length variation is universal and occurs in all classes [13]. The accretion of length variation as indels within a protein domain superfamily, however, was observed to be gradual and constituent domain members from the four major classes showed from <5 to >45% length variation (Figure 1). It was also observed that for domain superfamilies with at least 4 members, 20% of the domains showed over 30% length variation from the mean domain size. Where a majority of the members (>75%), show <10% or >30% variation, they were categorized as “length-deviant” (64) and “length-rigid” (24) domain superfamilies (Tables 1 and 2). This is not to imply that such domain superfamilies are populated exclusively by members showing extreme length variations, since length distributions in all ranges are universally observed in all classes.

**Table 1:** List of ‘length-deviant’ domain superfamilies.

S.No	Superfamily (SCOP Superfamily code)	PASS2 members	Av domain size	Std. Deviation	No of SCOP families	Super fold
**1**	**Concanavalin A-like lectins/glucanase (49899)**	26	197	38.5	21	Y
**2**	**SH3-domain (50044)**	14	71	15	1(38)	Y
**3**	**Translation proteins SH3-like domain (50104)**	5	100	33.4	6	Y
**4**	**GroES-like (50129)**	6	166	51.1	2(32)	-
**5**	**PDZ domain-like (50156)**	10	99	14.2	4(62)	-
**6**	**Bacterial enterotoxins (50203)**	13	99	13.7	2(23)	Y
**7**	**Nucleic acid-binding proteins (50249)**	39	112	37.9	14	Y
**8**	**Trypsin-like serine proteases (50494)**	30	225	34.8	4(99)	-
**9**	**ADC-like (50692)**	7	119	28	3(19)	-
**10**	**PK beta-barrel domain-like (50800)**	5	127	38.6	10	-
**11**	**Alpha-amylases, C-terminal beta-sheet domain (51011)**	12	78	15.9	5	-
**12**	**WW domain (51045)**	6	38	8.3	1 (10)	-
**13**	**Rudiment single-hybrid motif (51246)**	5	85	20.5	2 (11)	-
**14**	**E set domains (81296)**	42	122	31.4	20	Y
**15**	**(Trans)glycosidases (51445)**	46	360	67.4	14	Y
**16**	**Phosphoenolpyruvate/pyruvate domain (51621)**	5	341	122.5	6	Y
**17**	**Adenine nucleotide alpha hydrolases-like (52402)**	6	240	55.2	6	-
**18**	**Thioredoxin-like (52833)**	42	109	34.7	22	-
**19**	**P-loop containing nucleotide triphosphate hydrolases (52540)**	63	221	49	24	Y
**20**	**(Phosphotyrosine protein) phosphatases II (52799)**	12	234	59.7	5	-
**21**	**Aminoacid dehydrogenase-like, N-terminal domain (53223)**	7	153	34	5	-
**22**	**S-adenosyl-L-methionine-dependent methyltransferases (53335)**	21	238	38.9	52	-
**23**	**Nucleotide-diphospho-sugar transferases (53448)**	13	251	37	15	-
**24**	**Alpha/beta-Hydrolases (53474)**	39	354	97.4	35	Y
**25**	**“Helical backbone” metal receptor (53807)**	7	400	119.9	4(14)	-
**26**	**Periplasmic binding protein-like I (53822)**	13	376	79.4	1 (15)	-
**27**	**Periplasmic binding protein-like II (53850)**	15	255	55.7	2(69)	Y
**28**	**Thiolase-like (53901)**	12	193	54.4	2(22)	-
**29**	**Ankyrin repeat (48403)**	8	176	49.7	1 (16)	-
**30**	**Cysteine proteinases (54001)**	9	278	111.4	16	-
**31**	**Ribosomal protein S5 domain 2-like (54211)**	11	134	31.7	12	-
**32**	**FAD-linked reductases, C-terminal domain (54373)**	11	101	16.3	7	-
**33**	**MHC antigen-recognition domain (54452)**	13	143	45.8	1 (12)	-
**34**	**POZ domain (54695)**	6	95	19.9	2 (12)	-
**35**	**4Fe-4S ferredoxins (54862)**	8	95	36.7	6	Y
**36**	**Tetrahydrobiopterin biosynthesis enzymes-like (55620)**	7	155	38.4	4(15)	-
**37**	**C-type lectin-like (56436)**	22	120	19.7	8	-
**38**	**Acyl-CoA N-acyltransferases (Nat) (55729)**	10	194	57.3	9	Y
**39**	**Ferritin-like (47240)**	12	259	119.7	5	Y
**40**	**4-helical cytokines (47266)**	22	142	23.2	3 (38)	Y
**41**	**EF-hand (47473)**	35	125	40.4	11	-
**42**	**IHF-like DNA binding proteins (47729)**	6	76	22.9	2(8)	Y
**43**	**Terpenoid cyclase, Protein prenyl transferase (48239)**	6	308	71.3	4(10)	-
**44**	**ARM repeat (48371)**	9	369	222.8	22	Y
**45**	**TPR-like (48452)**	9	202	92	4 (23)	Y
**46**	**Carbohydrate-binding domain (49384)**	7	136	28.8	3 (11)	-
**47**	**p53-like transcription factor (49417)**	7	184	37.7	7	-
**48**	**Cupredoxin (49503)**	32	146	33.7	7	-
**49**	**Viral coat and capsid proteins (49611)**	31	227	73	9	-
**50**	**Cytochrome C (46626)**	22	101	24.3	8	-
**51**	**6 Phosphogluconate dehydrogenase C-terminal like (48179)**	6	191	86.7	12	-
**52**	**Viral proteins (49749)**	20	313	187	3(4)	-
**53**	**Rmlc-like cupins (51182)**	8	243	112.6	20	Y
**54**	**PRTase-like (53271)**	14	194	37	2 (39)	-
**55**	**Actin-like ATPase domain (53067)**	7	205	36.0	13	
**56**	**Homeodomain-like (46689)**	32	64	13.6	17	Y
**57**	**C-terminal effector domain of bipartite response regulator (46894)**	6	92	20.6	3 (13)	Y
**58**	**Putative DNA-binding domain (46955)**	5	90	20.4	7	-
**59**	**Histone-fold (47113)**	12	88	29.7	4 (38)	-
**60**	**Met repressor-like (47598)**	5	75	40.4	8	-
**61**	**Winged helix DNA binding domain (46785)**	48	88	20.7	68	Y
**62**	**NAD(P)- binding Rossman fold-domain (51735)**	49	183	39.7	12	Y
**63**	**Phospholipase D (56024)**	5	215	40.6	4(6)	-
**64**	**Lysozyme-like (53955)**	9	187	60.9	1(32)	-

[Standard deviations are good indicators of the extent of domain length variation but were not the sole criteria employed in classifying superfamilies as ‘length-deviant’ and ‘length-rigid’. The number of members in each superfamily that showed <10% or >30% length variation were also considered and classifications were based on trends in length variations for at least 75% of member proteins]. The SCOP code of each domain superfamily is provided in addition to the superfamily name in brackets. The number of SCOP families (number of structural protein domains is provided in brackets (in last-but-one column) when number of families <5) and superfold status of the domain is also provided (last column).

**Table 2:** List of length-rigid domain superfamilies (with at least 4 members).

S.No	Superfamily	Av_domain size	Av_Seq Id (%)	Std_deviation	No_families
**1**	**Cytochrome P450 (48264)**	417	21	31.4	1 (21)
**2**	**Terpenoid synthase (48576)**	323	14	24.6	5
**3**	**Nuclear receptor-ligand binding domain (48508)**	250	25	14.8	1 (32)
**4**	**DNA glycosylase (52141)**	204	23	18.4	6
**5**	**Calponin-homology domain, CH-domain (47576)**	114	26	9.6	1(9)
**6**	**TNF- like (49842)**	145	7	7.0	1(13)
**7**	**cAMP-binding domain-like (51206)**	135	29	3.2	3(13)
**8**	**C2 domain (49562)**	133	24	7.4	2(20)
**9**	**Actin-crosslinking proteins (50405)**	118	22	5.0	2(2)
**10**	**Invasin/Intimin cell adhesion fragments (49373)**	94	29	4.9	1(2)
**11**	**Sm-like ribonucleoproteins (50182)**	75	33	4.6	5
**12**	**ALDH-like (53720)**	474	23	32.6	2(15)
**13**	**Zn-dependent exopeptidase (53187)**	299	15	18.6	8
**14**	**Purine and uridine phosphorylase (53167)**	254	23	20.1	1(6)
**15**	**Metallo-hydrolase/oxidoreductase (56281)**	239	22	21.2	11
**16**	**Ribosome inactivating proteins (56371)**	253	30	8.2	2(17)
**17**	**Lactate and malate dehydrogenase, C terminal domain (56327)**	167	30	8.0	2(33)
**18**	**Superantigen toxins, C terminal domain (54334)**	111	36	5.1	1(14)
**19**	**UBC-like (54495)**	151	36	10.7	4(41)
**20**	**DNA clamp (55979)**	124	22	6.6	2(11)
**21**	**RNA-binding domain, RBD (54928)**	87	29	7.4	4 (73)
**22**	**Metal-binding domain (55008)**	70	36	2.5	1(8)
**23**	**Interleukin8- like chemokines (54117)**	70	38	8.9	1(24)
**24**	**Chromo domain-like (54160)**	67	33	5.2	3 (15)

It is observed that several domain superfolds that are repeatedly re-used in protein evolution in diverse domain architectures [17] are also found to be length-deviant (Table 1). Indeed, the propensity of superfolds to occur in length-deviant domain superfamilies is 1.9 as compared to length-rigid domain superfamilies (1.1) (data not shown). From the listing of the number of families in either dataset, it is clear that a number of length-deviant domain superfamilies have a large number of families suggesting that functional promiscuity may be anticipated. Indeed, indels are seen to impact either on the structure/function of these domains. Interestingly, single-membered domain superfamilies also retain the ability to invoke length variation and are also represented in length-deviant domain superfamilies (examples include SH3-domain like, GroES-like, WW domains, Ankyrin repeat, ADC-like domains *etc.*, Table 1). Likewise, length-rigid domain superfamilies are also represented by largely populated as well as single-membered domain families (Table 2). This suggests that the ability to accommodate indels is an intrinsic structural attribute of such domain superfamilies and is not solely a consequence of the structural plasticity of SCOP domain family members that belong to different superfamilies.

### A vast majority of the top length-deviant superfamilies exhibit structural repeats

Gene duplication is a method that facilitates evolution since it leads to the formation of phenotypically redundant genome portions that can be experimented for the generation of novel structural and functional products [2]. Domain repeats are considered a type of recombination in which two or more similar domains occur in tandem. In the course of evolution, all these forces play a vital role in increasing complexities involved in protein function and structural assembly.

Interestingly, eight of the top-10 length-deviant domains occur as structural repeats (Table 3 and Table S1). Full-length proteins from such domain superfamilies, include co-existing domain neighbors from the same SCOP superfamily since each repeat involves a duplication of the entire structural domain. We examined the domain assignments of full-length proteins for all length-deviant domain superfamily members to investigate the abundance of structural repeats. Further, the extent of repeat was verified through structural alignment methods such as DALI [18], LSQMAN [19] and also through the examination of domain topologies with HERA [20]. Short sequence repeats were also detected in online searches using the TRUST server [21]; however, in a majority of instances, these internal repeats were found to escape attention of simple sequence search procedures.

**Table 3:** Domain contexts in top-10 length-deviant protein domain superfamilies.

S.No	Superfamily	Single chain			Multi-chain			Domain repeats	Oligomers
		Single domain	Multi domain	Repeats	Single domain	Multi domain	Repeats		
1	Cytochrome C	17	4	-	5	1	6	Y	Y
Domain generally specified in a single/separate chains. In multi-chain proteins, other non-self domains may be specified by individual chains.									
2	SAM like domain	25	3	**-**	15	3	-	N	Y
Some members are multi-domain proteins and involve multiple chains. Repeats are not observed in this superfamily.									
3	6-phosphogluconate dehydrogenase C-terminal like	1	5	-	5	1	1	Y	Y
Usually a two-domain protein and involves the NADP-binding Rossmann fold. Members of Hydroxyacyl -CoA dehydrogenase protein family contain repeating copies of the structural domain.									
4	Viral proteins	-	-	2	-	-	3	Y	Y
Viral jelly roll, characteristic of this superfamily, repeats with varying lengths of interconnecting loops. These loops are involved in different subunit interactions.									
5	RmlC-like cupins	14	-	-	17	-	2	Y	Y
Includes members in diverse oligomeric arrangements and includes domains such as glycinins that have repeating copies of the entire cupin domain.									
6	Actin like ATPase domain	1	-	7	1	-	10	Y	Y
Tandem repeats of domain in a single chain are common. Members, all involving an ATP binding site, act on diverse substrates such as actin, glycerol kinase and hexokinase type I									
7	Phospholipase D	1	-	-	-	-	4	Y	Y
Giant members typically involve tandem repeats of entire domains. Dwarfs are single domain proteins that usually dimerize to function.									
8	PRTase-like	2	-	5	8	1	3	Y	Y
Diverse oligomeric states dictate an important role for loops of varying lengths.									
9	Lysozyme-like	6	-	-	3	1	1	N	N
Single domain protein on a single chain except for 1k28 (Tail associated lysozyme gp5), which has multiple domains involving multiple chains.									
10	Concanavalin A-like lectins	15	6	4	12	3	-	Y	Y
Most member proteins are involved in carbohydrate metabolism and occur as single domain proteins in a single or multiple chains. Multi-domain proteins interacting with 2–3 domains also exist.									
	**(%occurrence) in diverse domain contexts**	**36.6**	**8.0**	**8.0**	**29.5**	**4.5**	**13.4**		

We observe that domain repeats involving the entire structural domain can occur in single or multiple chains (Table 3 and Table S1). The domain assignments of ∼1200 proteins from 64 length-deviant domains (Tables S1 and S2) show that 27 out of 64 length-deviant superfamilies (42%) indeed form structural repeats as evidenced in at least one member of the superfamily. This number will likely increase with consideration of repeats in every domain superfamily member since only a representative structural member involving any one species for each protein domain family was considered here. Sequence homologues were not considered owing to large number of proteins to deal with and the possible decline in quality of the alignments. In a majority of the length-deviant domains analyzed, such structural repeats were appreciated with very good alignment scores (RMSD <2Å) involving 75% of domain length suggesting a duplication of the entire domain (Table S1). In protein domains such as protein tyrosine phosphatase II, flavocytochrome-C sulfide dehydrogenase (cytochrome C superfamily) and laminin (concanavalin A-like lectins), structural similarity is appreciable and covers ∼80% of the domains at RMSD <1.5Å. A few of these structurally repeating domains are also detectable at the sequence level. Indeed, the occurrence of structural repeats, as in topology of the domains, is likely to occur even more frequently as evident from 80% of the top-10 length-deviant superfamilies.

The number and lengths of repeats across different members varies across related members as seen in proteins that contain repeating copies of the TPR, ARM, Ankyrin repeats, EF-hand domains *etc.* (data not shown). In these domain superfamilies, differences in the number of structural repeats can generate varied interaction interfaces that confer additional functional properties amongst the different members. This is also observed in some larger domains such as the pectin lyase, cupins and domain superfamilies such as the four-helical cytokines that harbor diverse copies of the Ig-like fold.

Duplications of entire domains result in tandem arrangements of the self-domain along the length of the protein in a beads-on-a-string arrangement as seen in cupredoxins and phospholipase D or result in discontinuous arrangements of the domain as in squalene hopene cyclase. In the former type of structural repeat, dwarf members function as homodimers that associate to generate an active site. Giant domains meet such functional requirements by possessing multiple copies of the same domain on a single chain and most likely involve gene duplication events. Such tandem arrangements of domains involve longer loops that serve as linkers in bringing domains together and are seen in length-deviant superfamilies such as the phospholipase D, trypsin-like serine proteases, actin-like ATPases *etc.* (data not shown).

In length-rigid domain superfamilies, only 33% (8 out of 24) are engaged in structural repeats suggesting that internal repeats are more common in length-deviant domain superfamilies (Tables S1 and S3). We have restricted the current analysis to domain assignments of full-length proteins in structural databases and have not included sequence domain assignments, which would definitely complement and add to currently detected trends.

### Length-deviant superfamilies occur in diverse domain contexts

An important contribution to new structural interfaces/functional units is domain combination and shuffling resulting in new multi-domain architectures [22]. A majority of proteins are multi-domain, involving diverse neighbors (as co-existing domains) from different superfamilies. Indeed, domain combinations are important mechanisms in protein evolution [5], [23], [24]. We have examined the domain contexts of length-deviant domain superfamilies to examine the ‘social nature’ of such domains and their ability to associate with diverse domain neighbors. As seen in Table 3, ∼33% of the top length-deviant domains involve associations with multiple copies of either self or different domain superfamily in single or separate chains. This includes domains that occur as repeats, ∼21% involve multiple copies of the self-domain in single or separate chains. As reported previously, we also observe that it is more likely to have three or more repeats from the same domain family in tandem than fewer repeats (data not shown) [2], [22], [25].

Domain assignments were tabulated for 1189 protein domains from length-deviant superfamilies and 268 domains from length-rigid superfamilies. The recurrent domains have more domain partners. Of the 1189 protein domains that we have examined in the 64 length-deviant superfamilies (Table S2), 31.4% occur as truly single domain proteins. At least 26% occur as homologous domain copies in multiple chains. Additionally, such length-deviant domains are observed in multi-domain contexts in ∼42% of the protein domains examined, as opposed to length-rigid domains where only 22% occur in multi-domain contexts (Tables S2 and S3). The superfolds of NAD(P)-binding Rossmann domains, α/β hydrolase, SH3 barrel, OB fold and TIM domains are recurrent domain partners that are repeatedly employed as interacting partners of length-deviant domains from diverse domain superfamilies. It is interesting that such superfolds, that are themselves members of length-deviant domains, also find high representation as partnering domains.

### Length-deviant superfamilies have functional interactions with large numbers of protein domains

The diverse multi-domain contexts and multimeric states of length-deviant domains suggest that they are amenable to a variety of interactions involving different domain neighbors and that the range of interacting partners is extensive. To assess if this extends to functional interactions, for the top-10 length-deviant domains, we next examined the number of known and predicted protein-protein interactions in searches performed in the STRING database [26]. For every domain member, homologues with at least 60% sequence similarity were identified in *Drosophila*, yeast and other organisms and the number of known and predicted interactions was determined. We find that length- deviant domain superfamilies are highly interacting (1307), notably the domain superfamilies of SAM, cytochrome C and PRTase-like (Table S4). Such domain superfamilies are known to be functionally promiscuous and not only interact with diverse substrates, but are also regulated by a variety of proteins. They are also found in diverse domain contexts and occur in a variety of oligomeric states (Table S2).

Examinations of the length-rigid domain superfamilies showed that although ∼26% occur in multi-domain context and involve oligomeric interactions (Table S3), the type of domain neighbor is less varied with the same domain combinations reappearing across many members (data not shown). For instance, of the 24 length-rigid domain superfamilies examined, the members of 15 domain superfamilies have, at the most, one other partnering domain in the same polypeptide chain whose domain type is common across all the domain members and usually belongs to any one other domain superfamily. In these 15 domain superfamilies, the interacting domain type is conserved across all the domain members and usually belongs to any one other domain superfamily. The numbers of protein-protein interactions determined for such domains are also lower (798) (Table S4). Exceptions are observed for domains such as the calponin-homology domains and C2 domains. These domains are known to be structurally conserved modules involved in functional interactions with a variety of proteins.

### Functional implications of domain length variations

We have examined the contribution of indels to protein function in the 64 length- deviant domain superfamilies. In each domain superfamily, indels appear to be directly/ indirectly involved in a functional or a structural role. We discuss below some of these roles and strategies that are repeatedly employed by many domain superfamilies. A more detailed listing for the entire dataset is also provided in Table S5, where it is clear that indel roles are distinct and diverse in the different domain superfamilies. We expect that these roles are only likely to expand further with the inclusion and discovery of more protein domain superfamilies. Some length-rigid domain superfamilies show functional versatility as well (Text S1, Figure S1 and Figure S2). We also briefly discuss some of these strategies to highlight the various evolutionary approaches to mediate functional variety.

### 1) Additional lengths can confer extra thermal stability: Example of cytochrome C superfamily (SCOP code: 46626, S. No. 50 in Table 1)

**Figure 2:**
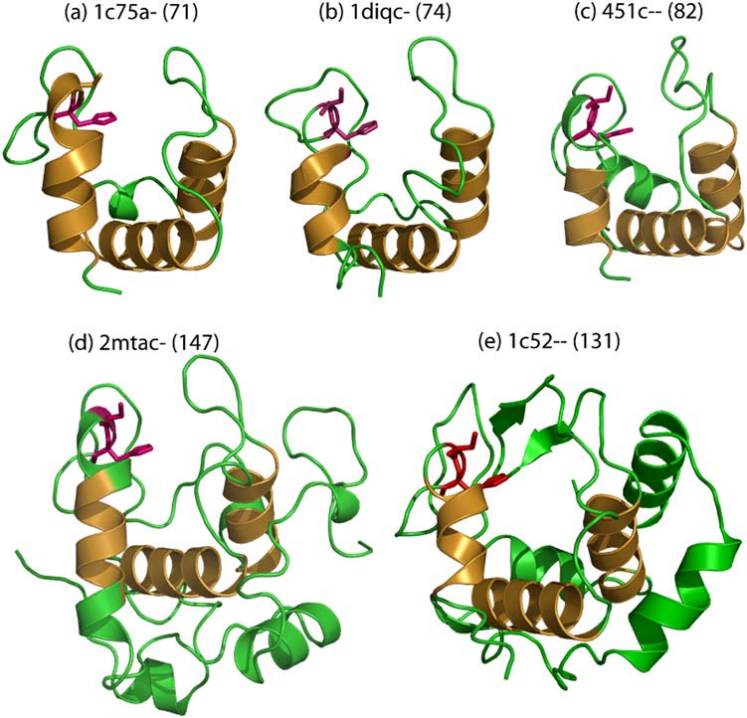
Members of the Cytochrome C- like domain superfamily (a–e) show two-fold length variation. Additional residues contribute to differences in the lengths of loops around the substrate-binding site. Cytochrome-C552 (1c52–: *Thermus thermophilus*) acquires two β-strands that further protects the bound-heme (not shown) from solvent. All structures preserve the hydrophobic pocket involving at least three helices (*shown in golden yellow*) surrounding the heme group (not shown).

The cytochrome C domain superfamily includes many proteins that are vital components of electron transfer mechanisms in both prokaryotes and eukaryotes. Diverse sequences (∼24% sequence identity) specify a characteristic fold (Figure 2) that consists of at least four α-helices around a heme group, a short 3_10_-helix and several turns. Domain superfamily members show up to two-fold variation in length (Table 4) (Text S1 for discussions on individual structural members in this superfamily). Manual examination of structural features of individual domain members shows that structural integrity of the heme-binding pocket with a heme-binding ‘CXXCH’ motif and a predominantly hydrophobic pocket, is well-conserved amongst all members [27] (Figure 2). In p-cresol methylhydroxylase, a flavo-cytochrome, a truncation of the cytochrome domain facilitates association with an additional flavo-protein domain. In cytochrome C-552, additional lengths are involved in a tight wrapping of the structure [28], [29]. Additional structural motifs in this domain superfamily are associated with distinct functional roles that appear to characterize each protein and even confer thermal stability to certain members. Most differences in length are due to variations in the lengths of surface loops connecting α-helices (Figure 2).

**Table 4:** Role of indels in top-10 length-deviant domain superfamilies.

S.No	Superfamily	Domain	Description	Role	Number of SCOP families
1	**Cytochrome C (46626)**	G: 1c52 (130)	Cytochrome C-552	**Thermal stability:** Two-fold length accretions occur predominantly in additional helices and long loops that pack tightly against the domain and bury the cytochrome deep into the structure.	**8**
		D:1c75 (70)	Cytochrome C-553		
2	**6-Phospho gluconate dehydrogenase- C terminal domain like (48179)**	G: 1pgj (299)	6-phospho gluconate dehydrogenase	**Dimer formation:** Additional length involved in dimer interface in the giant domain. The dwarf domain is truncated and sandwiched between an N- and C- terminal domain belonging to different superfamilies although a fair amount of structural similarity exists between the N- and C- terminal domain.	**10**
		D: 1dlj (97)	UDP-glucose dehydrogenase		
3	**Viral proteins (49749)**	G: 1p30 (631)	Adenovirus hexons	**Protein stability and size:** Each domain has indels (mostly loops) of varying lengths. These mediate interactions between the jelly roll subunits in these trimeric proteins.	**3**
		D: 1hx6 (139)			
4	**RmlC-like cupins (51182)**	G: 1pmi (439)	Phosphomannose isomerase	**Higher order complexes:** Cupin superfamily exists in diverse quaternary arrangements and such requirements may be facilitated by length changes.	**16**
		D: 1dgw (177)	Canavalin		
5	**SAM-like domain (53335)**	G: 1f3l (320)	Arginine methyltransferase	**Function regulation and specificity:** In the giant domain, additional lengths form a β-rich subdomain containing residues that interact with substrate and introduce functional specificity. Each member methylates specific substrates. In addition, it is implicated in an auto-regulatory role in the predicted biological dimer.	**41**
		D:1ej0 (179)	RNA methyltransferase (ftsj)		
6	**Actin-like ATPase domain (53067)**	G: 1bu6 (250)	Glycerol kinase	**Interdomain interface and substrate interactions:** All members carry out phosphoryl transfer involving ATP but act on diverse carbohydrates or include interactions between actin monomers. Additional lengths seen in helices and loops aid interactions with DNAse I or other actin monomers. In Actins, occur as N- terminal extensions to interact with other domains or involve in the interactions between substrate- binding residues and cofactors as in acetate kinase.	**11**
		D: 1j6z (142)	Actin alpha 1		
7	**PRTase-like (53271)**	G: 1ecf (242)	Glutamine phosphoribosyl transferase	**Substrate recognition:** Loops of diverse lengths lie in subunit interfaces and involve in diverse roles such as catalysis, allostery. Short loops are seen in dimeric PRTases since they lie adjacent to active site of adjacent subunits. Longer loops are often observed in monomeric PRTases. In addition, hoods of variable lengths recognize distinct substrates and are involved in specific reactions	**2**
		D: 1dkr (149)	Phosphoribosyl pyrophosphate synthetase		
8	**Phospholipase D (56024)**	G:1f0i (257)	Phospholipase D	**Multiple repeats:** Giant members possess two copies of the domain that relate in a pseudo-dyad symmetry. Longer loops pack the two domains together. Some loops may involve in enzyme interactions with membrane. Dwarf domains are functional dimers and possess shorter loops.	**3**
		D: 1byr (149)	Endonuclease		
9	**Lysozyme like (53955)**	G: 1qus (321)	Soluble lytic transglycosylase Slt35	**New interaction interface:** Additional residues may be involved in membrane interactions	**7**
		D: 1iiz (119)	Insect lysozyme		
10	**Concanavalin A-like lectins/ glucanases (49899)**	G: 1dyp (266)	Kappa carrageenase	**Quaternary interactions:** Carbohydrate recognition is mediated by loops of variable length in different members.	**21**
		D: 1slt (133)	S-lectin		

### 2) Variations in subunit interactions affect quaternary arrangement: Example of Viral proteins (SCOP code: 49611, S. No. 49 in Table 1)

**Figure 3:**
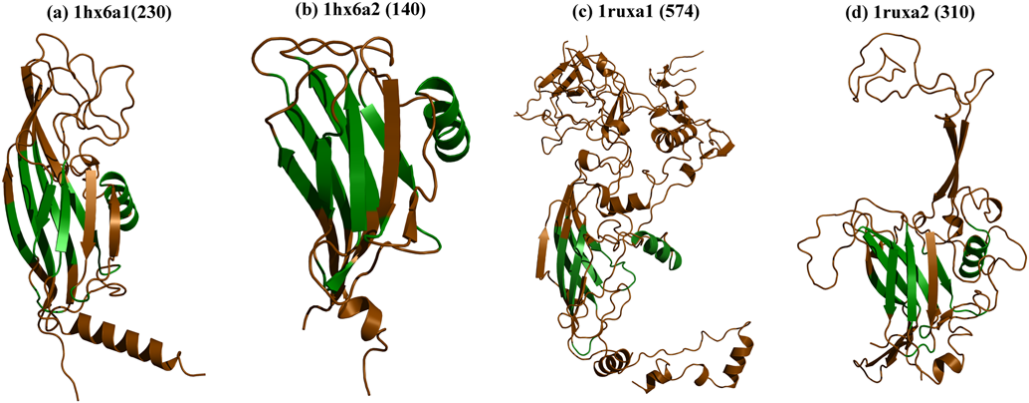
Domain members of the viral protein domain superfamily. A single subunit of the adenovirus type 5 hexon (1rux) and P3 of the bacteriophage (1hx6) involves two viral jelly roll domains (1ruxa1, 1ruxa2 and 1hx6a1, 1hx6a2 respectively). All four members show a conservation of the structural scaffold involving the viral jelly roll (*in green*). The nature of structural variations acquired by each domain (*in brown*) varies and loop lengths vary extensively even within a subunit. Additionally, residues in adenovirus (three-fold difference in length) form a subdomain involved in more extensive subunit interactions.

Protein domains that are involved in the coat and capsid proteins of viruses are rich in jelly rolls, well known for their huge length deviations and seen to adopt complex quaternary arrangements (Table 4, Figure 3, Text S1 for details). Capsid proteins often associate as homotrimers with three interlocking subunits, each subunit with two viral jelly roll domains. However, the association between the jelly roll domains differs across different members and results in distinct subunit interactions in each domain member [30]–[32]. Indeed, indels are seen primarily at such subunit interfaces and may ultimately dictate the size of the building blocks that form the viral capsid protein.

### 3) Domain duplication introduces functional diversity: Example of phospholipase D/endonuclease superfamily (SCOP code: 56024, S. No. 63 in Table 1)

**Figure 4:**
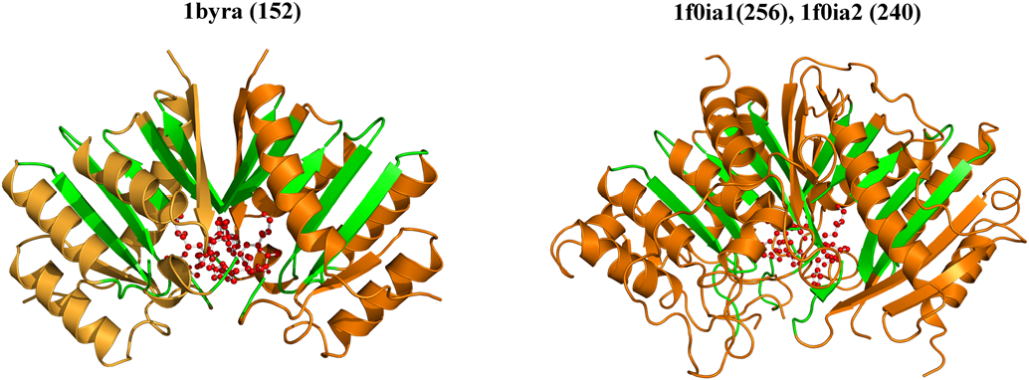
Structures of the giant and dwarf domain members of the PLD domain superfamily. Endonuclease (1byra-) and Phospholipase D (1f0i), the dwarf and giant domains of the PLD-like superfamily adopt different oligomeric states. Phospholipase D, a pseudo-dimer (1f0ia1: (256) and 1f0ia2: (240)), shows a duplication of the core domain of Endonuclease (1byra), which is a functional dimer. The PLD domain of endonuclease represents the minimum structural scaffold for acting on the phospho-diester bond of a substrate. The core conserved strands in either structure are highlighted in green. In endonuclease, residues from two HKD motifs (*in red*, *ball and stick*) from both protomers interact with the substrate. Phospholipase D has two copies of the motif and also shows some additional structures that protect the active site from solvent and move it deeper into the protein. Active site residues involve similar residues and lie in similar structural contexts (*in ball and stick*).

Diverse proteins such as the phospholipase D, cardiolipin synthases, phosphatidyl serine synthases, tyrosyl-DNA phosphodiesterase and endonucleases are members of this domain superfamily. Although each member acts on a distinct substrate, they are unified in their ability to bind a phosphodiester moiety in the active site for which they conserve, entirely or partially, two copies of an HKD motif to recognize the substrate (Table 4, Figure 4, Table S1, Text S1).

It has been suggested that the structure of the dwarf domain member, endonuclease, serves as the minimal structural scaffold for the hydrolysis of phosphodiester bonds and a gene duplication event may explain how the ancestral scaffold of endonucleases came to support an alternate function seen in the larger phospholipases [33], [34]. Such a duplication event in the larger phospholipases also results in a tandem arrangement of the domain repeat and ∼65% structural similarity between the repeating domains (Table S1).

### 4) Large length variations are required for substrate specificity and regulation of function: Example of S-adenosyl methionine dependent methyl transferases (SCOP code: 53335, S. No. 22 in Table 1)

**Figure 5:**
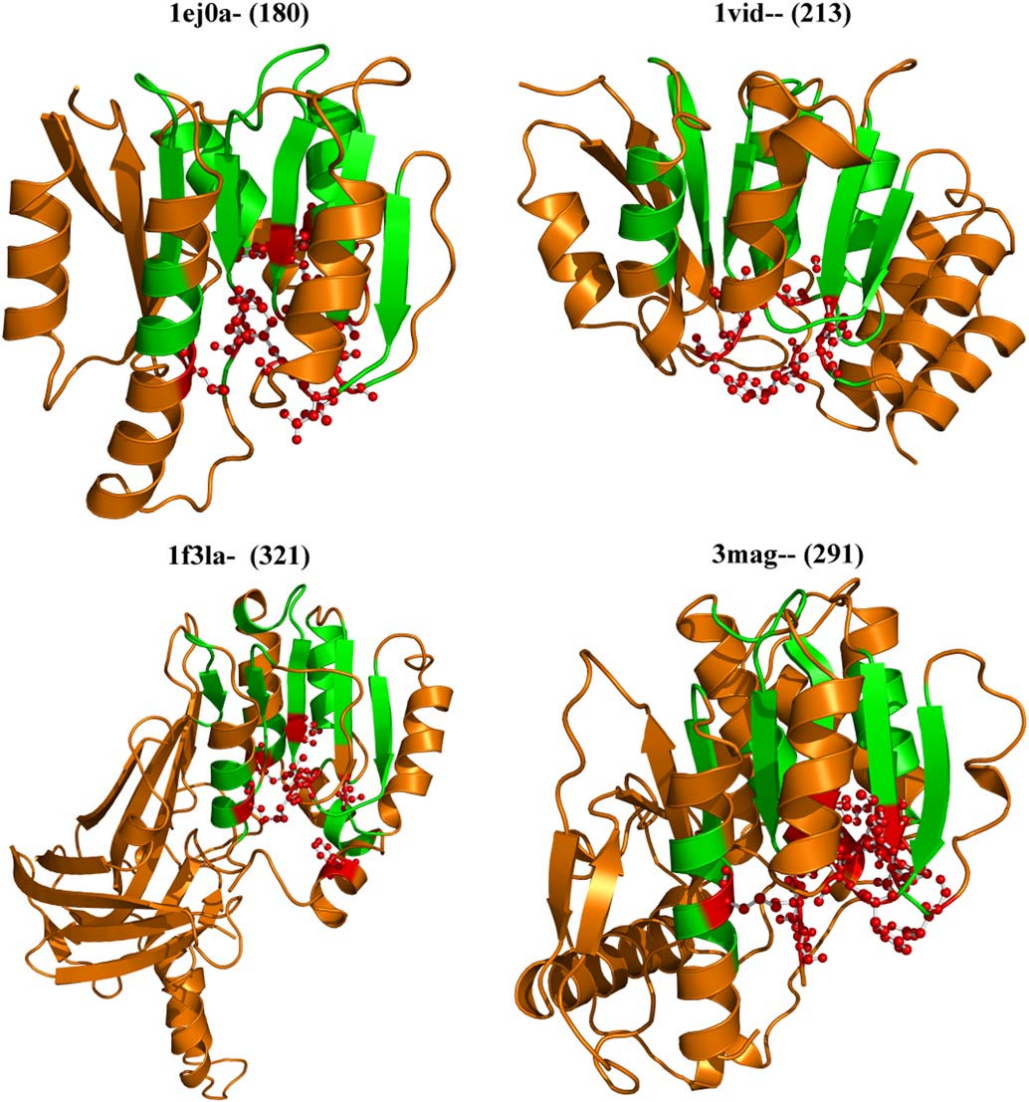
Domain members of the SAM domain-like superfamily. ftsj (1ej0a-), Catechol-O methyl transferase (1vid–), VP39 (3mag–), PRMT3 (1f3la-), show insertions that do not affect the common core structural scaffold (*in green*). Residues that interact with the Adomet cofactor (*ball and stick representation*, *in red*) and others that interact with the different substrates (not shown) are spatially proximate and their locations are conserved across the different members. In Vp39 (3mag–), a large 100-residue insert in the C-terminus appears to shield the core scaffold. In PRMT3 (1f3la-), the truncated SAM domain acquires a large barrel-like extension at the C-terminus. This subdomain-like indel contributes some residues to substrate-binding and may adopt an auto-regulatory role by interacting with Adomet binding residues of the neighboring subunit during dimer formation.

Biological methylation reactions that employ S-adenosylmethionine (S-Adomet) as the methyl donor are widespread and participate in a multitude of cellular processes through the methylation of a variety of substrates such as proteins, nucleic acids, phospholipids and small molecules. The domain superfamily includes ‘giant’ members such as the PRMT3 (321 residues) and VP39 (291 residues) and other ‘dwarf’ domains such as the ftsj and COMT that are only 180 and 213 residues in length, respectively (Figure 5, Table 4). The Adomet cofactor-binding residues are well-conserved. However, residues that recognize substrate differ in each member. As shown in Figure 5, the acquisition of additional residues in each domain member does not affect the core methyltransferase fold, but serves to introduce distinct substrate recognition features to each protein. Additionally, it also performs an auto-regulatory role in the largest of the domain members, PRMT3 [35], [36].

### 5) Additional lengths can generate new interaction interfaces: Example of lysozyme-like superfamily (SCOP code: 53067, S. No. 64 in Table 1)

**Figure 6:**
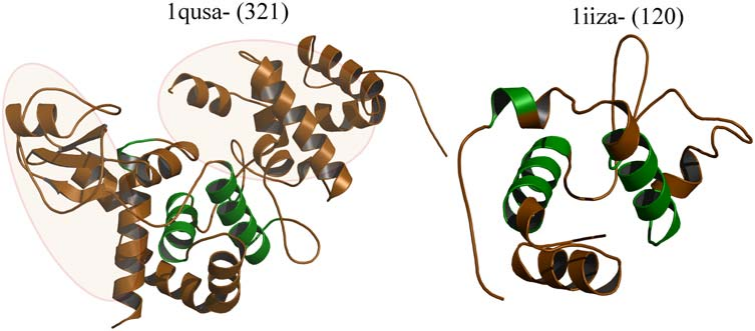
Lysozyme-like superfamily. Structures of lytic murein transglycosylase b (*1qusa-*, *321 residues*) and insect lysozyme (*1iiza-*, *120 residues*) show a well-conserved lysozyme-like fold (*in green*). Lytic murein transglycosylase acquires two additional N- and C-terminal subdomain like structures that are implicated in membrane interactions (*highlighted in faint pink*).

The lysozyme-like domain superfamily is a large multi-membered superfamily with at least seven different families in the SCOP database, all of them unified by the characteristic lysozyme-like fold. The ‘giant’ domain differs from other ‘dwarf’ domains of the lysozyme-like superfamily members in its acquisition of additional α-domain and β-domain extensions at its N- and C- terminal ends (Table 4, Figure 6). Additionally, extra length in this protein acquires an EF hand-like motif that may involve in the folding of the protein in the periplasm or in conferring increased stability [37]. Earlier structural analysis proposes that some of the residues in the α-domain might involve in anchoring the protein to the membrane [37] and thus present new interaction interfaces.

### Example of actin-like ATPase domain (SCOP code: 53955, S. No 55 in Table 1)

**Figure 7:**
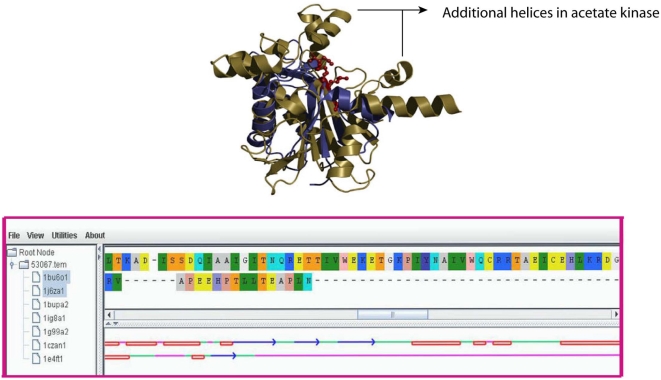
Actin-like ATPase domain superfamily. Superposed structures of acetate kinase (*242 residues*, *in gold*) and actin alpha1 (*142 residues*, *in blue*) show that the giant member acquires longer helices. The additional helical insert observed in acetate kinase forms a closed loop that brings residues that interact with the substrate close to the Mg^2+^ ion binding site. In other dwarf members of the superfamily, the same residues are involved in both ion-binding and catalysis thus obviating the need for such extra structural elements. The lower panel shows a graphical projection of the alignments. Large differences in length contribute to insertions of different structural elements in either protein (*Helix- red*, *strand – blue*, *coil – green*, *indels- magenta*).

The protein members of the actin-like ATPase domain superfamily include a varied set such as sugar kinases, heat shock proteins and actins that perform distinct functional roles involving phosphoryltransfer (Text S1). The range of length variation in this domain superfamily is almost two-fold and includes dwarfs such as actin (142 residues) as well as giants such as glycerol and acetate kinases (242 residues). Figure 7, shows a structural superposition of the C-terminal domains of the giant and dwarf domains of this superfamily. It is clear from the figure (and our graphical projection of the alignment) that the number and location of insertions varies between the members. The diversity of biological function within these domains appears to relate to different structural insertions that result in polymorphic loops and subdomains that connect the β-strands and α-helices in the core structure [38].

## Discussion

The collection of structure-derived domain superfamily alignments from PASS2 provides an opportunity to examine such alignments on a large scale in order to study domain length variations. Firstly, it has enabled a qualitative assessment of the extent of length variation in domain superfamilies and aided description of domains tolerant to length variation from a structural perspective. Secondly, by applying CUSP, we have examined the range of variations in diverse domain superfamilies by distinguishing structurally conserved blocks (of similar nature and lengths) from indels (regions susceptible to undergo length differences). We have found that the extent of length variation is not uniform across all classes. Thirdly, we have examined the role of such additional lengths in modifying/altering the general functions associated with a domain.Our investigations on the nature and typical lengths of indels showed that not all domains are uniformly tolerant to large variations in length and that certain domains are more susceptible. Indeed, of the 353 domain superfamilies considered, 64 domain superfamilies showed over 30% variation in length from their mean domain size.

In the present analysis, we have addressed the functional and structural advantages conferred on a domain due to indels by considering the extreme cases, namely the giant and dwarf in the length-deviant domain superfamilies. It is possible that large insertions, such as whole-domain insertions, have arisen in the proteins considered in our dataset and are actually due to large gene insertions and not pointing to subtle functional changes brought about by small length variations. However, our current dataset is not biased by such occurrences since we perform our analysis at the domain level and do not consider whole domain insertions. In every case, the functional roles were considered in the light of increasing domain sizes and the effect of loss of indels or decrease in average domain length were excluded since the direction of indel evolution is not the focus of the analysis. Earlier findings have already projected that long insertions predominate over long deletions that are also less likely to occur in protein domain evolution [39].

We have also consulted GO annotations for each domain member of the length-deviant domain superfamilies and find that 40 of the 64 length-deviant domain superfamilies include proteins that are involved in catalytic activity, where additional lengths are perhaps required to confer varying themes in substrate specificity (data not shown). At least 14 length-deviant domains are involved in regulatory processes and others are involved in structural roles where protein-protein interactions would be the main functional theme.

We find that in length-deviant domain superfamilies, additional lengths are associated with multiple roles such as substrate specificity, regulation, stability, generating interaction interfaces to form higher order complexes involving multiple domains in multimeric organizations *etc*. By an examination of the functional and structural advantages in these most length-deviant domain datasets, we determine, at least in outline, the different contributions that additional lengths confer on a length-deviant domain although more may emerge with the determination of new structures. The descriptions given here attempt to discuss the salient roles of extra lengths in the most length-deviant superfamilies but do not undermine the important contributions of shorter length changes in variant domains. Indeed, in domain superfamilies such as the lipocalins and DNA polymerases, incremental additions in lengths are associated with substrate specificity. We have also briefly examined those length-rigid domain proteins that are functionally versatile. The strategies employed here are refreshingly different and include changes in the orientation of structures, modifications local to the active site to attain functional diversity despite such high structural integrity (Text S1 and Figure S1 and Figure S2).

We have also investigated whether length-deviant protein domains associate to form higher order complexes. In length-deviant superfamilies, nearly 40% of length-deviant domains function as multimers and involve interactions with variable copies of self or other domains. Although in the current analysis, we did not find any statistically significant correlation between such trends in length-deviant and length-rigid protein superfamilies (∼30% length-rigid proteins also do function as multimers), we believe that this is a consequence of the high variability in the number of proteins in each dataset. Length-rigid protein domains are not as well-populated (270) as the number of length-deviant domains (1130) and this could affect the numbers projected for length-rigid domains. Here again, although the data is not discussed, we have observed that the number of interacting partners in length-deviant domains is far more than the length-rigid proteins and a more in-depth analysis is required to understand why this may be the case.

The interesting trends that we have obtained on the nature and type of indels in protein superfamilies from different classes could affect the area of comparative modeling in structurally unconserved regions in newer superfamily members. Our analysis has shown that in a majority of the superfamilies that we have examined, the core structural scaffold is rarely affected, despite length differences. However, even within a superfamily, the extra lengths impacted differently on function for different members, and therefore, it may be difficult to generalize the exact role of additional lengths in newer members. Depending on their locations and lengths in the structure, we may be able to suggest an involvement in introducing substrate specificity, or in presenting newer protein interfaces for interaction with other proteins or promoters.

What is the wealth component that dictates such vivid length variations observed in some protein superfamilies? We find that the ‘currency’ for versatility in length of domain superfamilies is not differential amino acid composition since both length-rigid and length-deviant domain superfamilies exhibit similar amino acid propensities. Could the complexities of domain architecture, nature of co-existing domains, need for internal symmetry, repeating structural themes and diverse quaternary arrangements dictate length variations amongst related protein members of a superfamily? Our analysis suggests that a multitude of these parameters operate to influence the structural revolts of length-deviant domains, imposing still a daunting exercise to predict such variations.

## Materials and Methods

### A library of protein domain superfamilies that show length differences

In the current study, we have employed SCOP [9] domain definitions that consider domains as fundamental evolutionary units capable of existence in isolation or in association with other domains. SCOP groups related domains with high identities into a family and into superfamilies, those proteins with evolutionary features dictated by common features of structure, function and sequence. The PASS2 database [40] contains structure-based SCOP domain superfamily alignments (version 1.63) that have been derived using COMPARER [41] and STAMP [42].

We have considered 353 multi-member superfamily alignments (with at least 3 distantly related members) from PASS2 [40] (where the sequence identity between any two members in a superfamily is not more than 40%) and determined the extent of length variation in each domain superfamily. For this purpose, the mean domain size for each domain superfamily was determined by averaging domain lengths of individual members. The length difference of each member was then expressed as a fraction of the mean domain size. Additionally, in methods described in detail elsewhere [13], standard deviations in length from the mean domain size were also calculated for each member using standard formula and averaged for the entire superfamily. Thus, each domain superfamily was associated with a range of length variation exhibited by its constituent members. The distribution of length difference for each member over different length ranges was plotted. For domain superfamilies with at least 4 members, 20% of the domains showed over 30% length variation from the mean domain sizes and were grouped as ‘length-deviant’ domains. Domain superfamilies, where 75% of domain members show <10% length variation from the mean domain size, were grouped as ‘length-rigid’.

We have also applied the CUSP algorithm [13] on each of the 353 domain superfamily alignments to determine the locations, typical lengths and preferred structural types of length insertions amongst related domain superfamily members. CUSP examines a domain superfamily alignment and internally maps DSSP and PSA scores to each member sequence. It scans each alignment position and employs a scoring scheme, tested on diverse datasets, to detect structurally conserved regions observed in all domain superfamily members and distinguishes such regions from indel regions where differences between members in terms of length or structural type accumulate.

### Algorithms employed for detecting internal repeats and domain duplications

To examine the occurrence of structural repeats in domain superfamilies, giant and dwarf domains were identified in each length-deviant and length-rigid domain superfamily. Full-length protein sequences for each of the giant and dwarf domains were retrieved from the SWISS-PROT database [43]. Each full-length sequence was queried against the HMM models of domain superfamilies available in the SUPERFAMILY database [44]. It is considered that if at least two domains in a sequence are assigned to the same superfamily, the presence of a structural repeat is implied. Further, their presence and location in related domains was checked with topology diagrams using HERA [20]. Additionally, these repeating domains were also aligned using DALI [18] and LSQMAN [19] to appreciate the extent of structural repeat. For the same full-length sequences, the presence of internal sequence repeats was also assessed through searches in the online TRUST server [21].

### ‘Domain contexts’ of length-deviant domains

We define ‘domain context’ as the preferred mode of occurrence of a domain. Domain superfamily members differ in their associations. We attempted a correlation of the observed length variations with domain contexts and nature of domain associations for each length-deviant and length-rigid domain superfamily. For each length-rigid and length-deviant domain superfamilies in our dataset, all domain members were pooled together. If a domain member is available from multiple species, a representative sequence with the best resolution from any one species was selected. Full-length protein sequences were obtained from the SWISS-PROT database for representative domains from any one species. SCOP domain assignments were made for each sequence. Full-length proteins of known crystal structure were considered for domain assignments and structural databases alone were consulted for this preliminary analysis. In addition, the occurrence of domains singly (single domain in a single chain or single domain in multiple chains), repeating domains (multiple copies of a domain i.e., domain repeats in a single or multiple chains) and in their domain associations (single/multiple copies of a domain in association with neighboring/partnering domains in a single or multiple chains) was also noted.

### Functional interactions of domains

Functional interactions of length-deviant and length-rigid domain superfamilies were studied by examining the known and predicted protein-protein interactions for each domain member in searches in the STRING database [26]. More than 80% of the proteins in the test set show >60% sequence similarity with the proteins in the STRING database and the lowest level of similarity observed between the test set and entries in STRING was 40%. Further, to determine if domain superfamilies that are length-deviant/rigid are of specific functional types, GO annotations were derived for each domain superfamily member through an online submission of domain sequences in FASTA format to the GOAnna server (unpublished). GOAnna employs BLAST sequence similarity search to derive GO annotation terms for the closest sequence homologues of a query sequence. The annotations for each member were examined manually to determine trends, if any, in length-rigid and length-deviant domain superfamilies.

### Role of indels in domain function

We have analyzed the functional roles of indels for the 64 length-deviant domains by examining if indels are involved directly or indirectly in domain function in the giant (longest) and dwarf (shortest) domains of each length-deviant domain superfamily. Indels were identified by the CUSP method and alignments were projected through a graphical viewer, StructView, described elsewhere [13]. For every giant and dwarf domain member of each domain superfamily, the involvement of indels in protein function was determined by consulting literature, where relevant, and by manually examining protein structures to determine the proximity of indels to functional sites or sites involved in protein-protein interactions. We have also examined length-rigid superfamilies, in a similar manner, to appreciate better their diverse functions in the light of a strictly conserved domain size.

## Supporting Information

Text S1 Functional variety in deviant and rigid domain superfamilies. This text file provides detailed description of eight types of functional attributes to length-deviant superfamilies and five superfamilies of length-rigid superfamilies by giving relevant examples.
(0.14 MB DOC)

Figure S1 DNA-glycosylase domain superfamily. The two domain scaffold of the DNA-glycosylase domain superfamily in Adenine glycosylase and Endonuclease III harbors a HhH motif (in pink) with active site residues (in red) to bind their respective substrates. Composition of residues in the active site is distinct for each member.
(0.51 MB TIF)

Figure S2 Interleukin-8-like superfamily. Interleukin-8 like chemokine superfamily shows high conservation of the core structure. Lymphotactin (a) and stromal cell derived factor 1 alpha (b) differ primarily in the N and C termini. Lower panel (c) shows a graphical projection of the alignments. The core structure involving the well conserved 310 helix and the three stranded sheet is well conserved across different members and structurally equivalent regions in the alignment are extensive.(Helix- red, strand - blue, coil - green, indels- magenta)
(0.15 MB TIF)

Table S1 Structural repeats in length-deviant and length-rigid protein domain superfamilies
(1.27 MB DOC)

Table S2 The number of occurrences of domains singly, multiply and as repeats (tandem repeats of domain) in a single or multiple chain are provided.
(0.31 MB DOC)

Table S3 The number of occurrences of domains in single(SD), multiple(MD) and as R (tandem repeats of domain) in a single or multiple chain are provided. R+MD includes domain occurrences that show repeating copies of self domain in multidomain contexts.
(0.07 MB DOC)

Table S4 Number of Protein-protein interactions (known and predicted) in length-deviant and length rigid domain superfamilies after searching in STRING databaseS30.
(0.05 MB DOC)

Table S5 Structural and functional role of indels in the 64 length-deviant domain superfamilies
(0.05 MB XLS)
