# Supplementary material for: Length Variations amongst Protein Domain Superfamilies and Consequences on Structure and Function
Source: PLoS One. 2009 Mar 31;4(3):e4981. doi: 10.1371/journal.pone.0004981 (PMC2659687; doi:10.1371/journal.pone.0004981)
Supplement: Text S1 — Functional variety in deviant and rigid domain superfamilies. This text file provides detailed description of eight types of functional attributes to length-deviant superfamilies and five superfamilies of length-rigid superfamilies by giving relevant examples. (0.14 MB DOC) [file pone.0004981.s001.doc]

**Text S1:**

**Length variations amongst protein domain superfamilies and consequences on structure and function**

Sankaran Sandhya^1^, Saane Sudha^1^, Barah Pankaj^1,2^, Madabosse Kande Govind^1^,

Bernard Offman^3^, Narayanaswamy Srinivasan^4^ and Ramanathan Sowdhamini^1*^

**Functional variety in length-deviant domain superfamilies**

**1) Additional domain lengths can confer extra thermal stability**

**Cytochrome C:**

The cytochrome C domain superfamily includes domains that show over two-fold variations in length. In the dwarf domain of P-cresol methylhydroxylase (PCMH), a flavocytochrome C, the cytochrome subunit is truncated with only three α-helices. Such a ‘domain dwarfing’ may be due to its two-domain context that contains a flavoprotein subunit in addition to a cytochrome domain ^S1^. The tight association between the domains may result in a ‘dwarfed’ cytochrome subunit with nearly 20% of the cytochrome structure involved in inter-subunit interactions with the flavoprotein subunit. Such dwarfing is observed in other length-deviant proteins such as the protein methyl arginine transferase-3 that exists in multi-domain context and may explain the diverse lengths adopted by deviant domains. In contrast to this, cytochrome C-552 (131 residues) from *Thermus thermophilus* is a giant member having eight α-helices that is similar to other cytochromes in regions involving the heme prosthetic group^S2^ (Figure 2). However, the additional residues in this protein are involved in structures that appear to confer thermostability. The wrapping of the structure by the longer clamp-like C-terminal one-third of the molecule bound tightly by hydrophobic contacts and salt bridges to the rest of the protein and the accommodation of the additional residues in the extended β-sheet near the heme both serve to confer such advantages to these proteins^S2^ (Figure 2). In most members, the heme group is enveloped by a core α- helical structure that is shielded from the solvent by coils of varying lengths.

Thus, length variation in this protein superfamily appears to address the different physiological requirements of this protein and plays a vital role in shielding the substrate from solvent.

**2) Variations in subunit interactions affect quaternary arrangement: Viral proteins**

In each subunit, the two viral jelly rolls are 230 and 140 residues in length respectively. While the two viral jelly rolls (V1 and V2) are similar in topology, they differ in the number and lengths of connecting loops. The differences between V1 and V2 within each subunit relate to the different contacts that each makes with other subunits in the final assembly of the homo-trimer. In structural comparisons of the jelly rolls V1 and V2, it is found that 90 additional residues in V1 are observed in loops ^S3^. These loops are involved in contacts with similar loops from neighboring subunits and form tower-like extensions from the capsid surface. The base of each subunit also supports a long helix from V1 that extends beneath the jelly roll and is implicated in interactions with the membrane ^S3^.

Another related domain in the superfamily is that of the capsid proteins from human adenovirus type 2, hexon. These domains also form homo- trimers and possess similar overall architecture as the P3, except that they are ~900 residues in length. As shown in Figure 3, like P3, hexon also contains two viral jelly rolls, P1 and P2, arranged in an orientation similar to V1 and V2 in P3. Both P1 and P2 (574 and 310 residues) are longer than V1 and V2. Domain comparisons show that additional residues in hexon contribute to the far more extensive loop arrangements around P1 and P2^S4^. A 17-residue loop specifically involved in subunit interactions in V1 jelly roll of P3 is 191 residues long in the P1 jelly roll of hexon. Similar length differences are noted in the other loops that pack the subunits close together^S3^. The extensive loop arrangements in hexon produce a larger building block required to form a larger virion (920 Å) than PRD1 (740Å) and are, thus, crucial for the precise assembly of the icosahedral viral coat.

**3) Additional domain duplication introduces functional diversity: Phospholipase D**

Phospholipase D possesses hydrolytic activity and participates in the synthesis of low abundance phospholipids by catalyzing transphosphatidylation reactions in the presence of alcohol. The structure of Phospholipase D contains two tightly interacting and similar N- and C- terminal domains (256 and 240 residues respectively), which relate by a pseudo-two fold rotation axis running through the active site-bound phosphate ^S5^ (Figure 4). Each domain contains an HKD motif and catalysis proceeds through interactions involving residues from both motifs. The overall structure of Phospholipase D is similar to the dimeric structural assembly of Endonucleases that are dwarf domains (152 residues) of this family from *Salmonella typhimurium* (Table 4). Endonucleases, hydrolyze double and single-stranded DNA, and possess only a single conserved HKD motif ^S6^. The pre-requisite for an interaction between two HKD motifs for catalysis however, is satisfied through the enzyme functioning as a dimer, unlike in other members. The active site is formed through the union of two HKD motifs from both protomers.

As seen in Figure 4, the secondary structural elements are more tightly packed with shorter connecting loops in endonuclease. Endonuclease that can act on single and double stranded-DNA has a more open active site since it is less specific for its substrate. In phospholipase D, additional length is seen in longer loops that act as lids to shield the active site or additionally aid in embedding the enzyme into the membrane ^S5^.

**4) Large length variations are required for substrate specificity and regulation of function: S-adenosyl-L-methioninedependent methyl transferases**

This superfamily of proteins adopts the canonical S-AdoMet-dependent methyltransferase fold and includes a diverse array of proteins, which not only act on different substrates such as the rRNA-binding heat shock protein (ftsj) and vaccinia mRNA 2’O-methytransferase (VP39), the protein-binding glycine n-methyltransferase and protein arginine methyl transferases (PRMT3) but also small molecule-binding catechol-o-methyl transferase(COMT). Figure 5 shows the typical methyltransferase fold that consists of a seven stranded β-sheet flanked by five α- helices as seen in ftsj. In COMT, at least 8 α-helices flank the seven-stranded β-sheet. In Vp39, which specifically recognizes N7-methylguanine moiety of the mRNA cap, the core structure harbors an additional 100 residues in the N- and- C terminal helices and loops to occlude a face of the core and creates a pocket for the binding of the substrate ^S7^. Although a larger protein, PRMT3, has a truncated methyltransferase domain with a five-stranded β- sheet that appears to compensate for a longer C-terminal barrel-like extension. In addition, it possesses two short additional N-terminal α- helices that position themselves near the active site. The long subdomain-like barrel contributes a three-helical arm that interacts with the AdoMet-binding domain of neighboring molecules in dimeric assemblies of this domain. It is speculated that the additional barrel like domain of this protein, controls the binding of AdoMet and thus regulates the activity of PRMT3, which is to recognize and cleave RXR clusters in substrate proteins. Experiments showing that these enzymes show higher activity for bound peptides than for larger protein substrates suggest that such additional structures enclose the active site into a hole, thereby restricting the size of the substrate ^S8^.

**5) Additional lengths can generate new interaction interfaces: lysozyme-like superfamily**

Dwarf domains, such as the chicken lysozyme [C-type lysozyme] and insect lysozyme, are structurally and functionally similar (129 to 120 residues, respectively)^S9^. Although both lysozymes exhibit comparable antibacterial activity against Gram-positive bacteria, their physiological roles differ and the insect homologue is induced on bacterial infection while the chicken lysozyme is constitutively expressed. This difference translates to the minor length modifications between these protein structures and additional residues occur in loops of the chicken homologue. Structural studies^S9^ suggest that the truncation in loops in the insect protein may affect its half-life by increasing the thermal stability of the protein, a much-required feature for an induced protein, unlike the constitutively expressed chicken lysozyme. Larger domain relatives, such as the lytic transglycosylases, function as bacterial muramidases, which catalyze the cleavage of the β-1,4-glycosidic bond between N-acetylmuramic acid and N-acetylglucosamine^S10^. Lytic transglycosylases may either be water soluble such as Slt70 [168 residues] or membrane-bound, like murein transglycosylase [Mlt A-D]. Slt35 [321 residues] is a naturally occurring soluble fragment of the membrane bound MltB. Thus, domain members of this superfamily seem to have acquired additional extensions at the N- and C-terminal ends, the exact functional roles of which are unclear since neither of the subdomains at the N- and Cterminal ends resembles any known structure.

**6) Additional lengths in domains are involved in dimer formation:**

**6-phosphogluconate dehydrogenase C-terminal domain-like superfamily**

The C-terminal domain of UDP-glucose dehydrogenase from *Streptococcus pyogenes,* a dwarf protein with only 98 residues and that of 6-phosphogluconate dehydrogenase from *Trypanosama brucei* (300 residues), which is nearly twice as large, are two members of this superfamily (Table 4). Although these two proteins act on very different substrates, structural superposition of the two C-terminal domains shows a remarkable conservation in identity and conformation of two of the active site residues that co-ordinate NAD. Manual examination of the superposed proteins shows that the similarity between the two proteins involves, primarily, the common N-terminal NAD^+^ binding domain shared by both proteins^S11^. This similarity extends partially to a predominantly α-helical region in the C-terminal domain of either protein. In UDP-glucose dehydrogenase, this domain consists of a long α- helix that lies at the interface of the N- and C-terminal domains of the protein. The C-terminal domain of this protein is almost identical to the N-terminal dinucleotide-binding fold resulting in an inter-domain pseudo-symmetry of the dinucleotide-binding fold. It is also the domain primarily responsible for binding the UDP moiety of the UDP-sugar. In 6-phosphogluconate dehydrogenase, a functional dimer, such a pseudo-symmetry and duplication of the N-terminal domain is not observed. Instead, the eleven ‘α- helical’ C-terminal domain is longer [300 residues], forms a part of the dimer interface and contributes a few charged residues from its C-terminal tail to interact with the co-enzyme^S12^ in the other subunit.

**7) Additional lengths are involved in substrate recognition**

**PRTase-like superfamily**

Proteins such as xanthine-guanine phosphoribosyltransferase, orotate phosphoribosyltransferase, hypoxanthine-guanine phosphoribosyl transferase, glutamine phosphoribosyltransferase and other type I PRTases that adopt this canonical fold are characterized by a sheet of at least five β-strands, three or four α-helices and a 13-residue sequence motif. The core fold is expanded by two to five additional structures in related members (Table 4). Three predominant loops occur in the core PRT structure of which the PPi and PRPP loops interact with the bound PRPP group in all PRT members.

The core scaffold is well–preserved in all the structures, however, distinct insertions are made in each structure. These usually involve changes to the flexible loop that has strikingly different lengths in different PRT proteins and yet, shields the substrate from the solvent. In many PRT proteins, the length variations occur at subunit interfaces and contribute to Mg.PrPP binding in two subunits. This flexible loop plays an essential role in the specific biological function of each PRT, which includes catalysis, allostery, inter-domain signaling and channel formation. Shorter loops are seen in the dimeric OPRTs since the loop is spatially proximate to the active site of the adjacent subunit while longer loops are seen in most other PRTases. Above the core fold, PRT proteins also have a hood of variable length contributed by both N- and C-terminal residues that play different roles in each member. In some, such as the pyrimidine PRTases, they are involved in recognition of the appropriate base, while in PRPP synthetase, they are involved in the generation of PPi from ATP^S13^.

**Actin like ATPase domain**

Whereas sugar kinases, glycerol kinases and acetate kinases catalyze the uptake of carbohydrate substrates into metabolism, others such as heat shock proteins [Hsc70] are central elements in protein folding and actin are ubiquitous structural and contractile proteins. An unifying element in this superfamily is that functional roles are driven by the binding of ATP in the presence of Mg^2+^ or Ca^2+^ ions and always involve phosphoryl transfer.

Despite differences in lengths and poor sequence similarities between the proteins, the intact proteins of all superfamily members usually contain two domains (N- and C- terminal) that share a dyad symmetry that might have evolved through a gene duplication event^S14^. All members conserve a fingerprint sequence of the nucleotide-binding pocket which occurs at a cleft between the N- and C-terminal domains, except in hexokinases where ATP binds only one of the domains^S14^.

Examination of secondary structures (data not shown) between related domains shows that structural variability in terms of secondary structure composition occurs primarily in helices (~54%, 47% and 29% in helical, strand and coil regions).

In actin, such regions are involved in interactions with other actin monomers or in binding the myosin heavy chain subfragment 1. Some other loops that form a subdomain in actin (not shown in figure) are involved in contact with Dnase I. In hexokinase (207 residues), an N-terminal extension is used to make an additional interdomain connection not seen in the other structures. In acetate kinases, a helical insert (highlighted in Figure 7) forms a closed loop structure that brings the substrate-binding residues in close structural context with the Mg^2+^ ion binding residues^S15^.

**8) Additional lengths are involved in diverse quatenary contexts**

**Rmlc-like cupins**

The cupin superfamily of proteins adopts an ancient fold that is universally represented in all the three kingdoms. Indeed, this superfamily includes not only strict mono-domain cupins but also bi- and multi-domain members, each exhibiting a different degree of preferred oligomerization. The differences in inter-motif length that varies from 11 to over 100 residues in the different members, defined within the structural core, are implicated in the diversification of functions in this superfamily and addition of residues to the N- and C-terminal ends in the giant members are believed to contribute to the different multimeric structures seen in this superfamily. Repeating domains in members like proglycinin show appreciable structural overlap (Table S1) comprising ~57% of the domain. The selection of multiple roles for this primitive scaffold is often attributed to the high thermal stability that this fold offers through maximizing inter-subunit contacts, hydrophobic interactions, hydrogen bonding and efficient packing through shorter loops and fewer cavities^S16^. Interestingly, all members retain a common β-barrel scaffold of two well-conserved strands separated by a less conserved region of two other β-strands with an intervening loop of variable length.

**Concanavalins**

Likewise, a length-deviant domain superfamily (containing canavalins and lectins) involved in carbohydrate recognition also occurs in diverse quaternary states and involves repeating domains (Table 4 and Table S1). Loops of different lengths are implicated in the recognition and binding of distinct substrates^40^. Higher levels of specificity in recognizing multivalent carbohydrates are affected by the quaternary contexts of these domains further influenced by loops of varying lengths.

**Functional variety in length-rigid domain superfamilies.**

Since it was observed that the extent of length variation is distinct for each superfamily, we have delineated the multi-member PASS2 domain alignments into ‘length-rigid’ and ‘length-deviant’ superfamilies. In many length-deviant domains (examples discussed in main text), additional lengths in indels were seen to directly or indirectly involve in domain function or in domain interactions with neighbours.

~40% of 353 domains in our dataset are ‘length-rigid’ with domain size conforming to an average size. Such length-rigid domains are not as well-populated as length-deviant domains where at least 64 domain superfamilies have >4 members. The number of multi-membered superfamilies (>4 members) in our length-rigid dataset is only a quarter of the deviant domain dataset making comparisons between length-rigid and length-deviant domains difficult. Yet, the functional variety and interactions of length-rigid domains *vis-a-vis* length-deviant domains suggests that such rigid scaffolds adopt alternate strategies to achieve promiscuity in function. To address this, we have examined a few length-rigid domains that are also functionally versatile by manually examining the structural alignments and location of functional residues in such proteins. In addition, we have also analysed the various domain contexts and associations of such domains.

**‘Rigid’ domain superfamilies and functional variety**

**1) DNA-glycosylase**

Members of the helix-hairpin-helix DNA-glycosylase superfamily include an interesting range of proteins that repair lesions on DNA. Each member recognizes a specific type of lesion, which may result from mismatch mutations or the presence of oxidized and alkylated bases. Whereas, endonuclease III removes modified thymine and cytosine bases from DNA^S17^, adenine glycosylase, specifically binds modified adenines on DNA^S18^. 3-methyladenine DNA-glycosylase, another member, removes alkylated purines^S19^. Each enzyme exhibits a clear preference for a distinct substrate. All three members, maintain structural integrity and involve a HhH motif in binding their different substrates. Figure S1 shows the two-domain scaffolds of Endonuclease III and Adenine glycosylase with active site residues located on the HhH motif between the two domains. While the three enzymes conserve the location of the active site pocket, they exert a clear preference to line the pocket with chemically distinct residues. Endonuclease III, for instance, possesses a polar active site pocket containing water molecules to recognize damaged pyrimidines while 3-methyladenine DNA-glycosylase enriches its active site with aromatic residues. Adenine glycosylase, on the other hand, retains a rich assortment of positively charged residues to recognize its modified DNA^S20^. The structural alignments in the PASS2 database^S21^ for this superfamily show that a strong structural equivalence exists over much of the protein alignment and small variations in length between members occur away from the active site pocket. Diversity in specific function seems to be accommodated through differences in sequence composition at active sites and structure and length of the members are well-preserved.

**2) Interleukin-8 like chemokines**

Many different proteins such as lymphotactins, macrophage inflammatory protein 3-alpha, fractalkaline etc., are members of the interleukin-8 like superfamily. These proteins show considerable uniformity in sequence length with a typical domain size of nearly 70 residues. Examination of the structural members of this superfamily shows that despite limited sequence homology, all members show a high degree of structural similarity in their protein core (Figure S2). This consists of three β-strands and one α-helix in the C-terminus. All chemokines also strictly conserve cysteine residues in the N-terminus that confers a stable fold through the formation of disulphide bonds. However, sequence and minor loop length variations involving residues located at the N-terminus up to the start of a 3_10_ helix, which precedes the core structure, are actively involved in the specific recognition of these proteins by their respective G-protein coupled receptors^S22^. As seen in the alignment (Figure S2), structural equivalence is appreciable along the alignment length and most differences between members lie in N- and C- terminal ends that are involved in recognition of substrates.

**3) Calponin-homology domain superfamily**

Many actin-binding proteins such as calponin, fimbrin, spectrin, utrophin etc., possess a ~100 residue structurally conserved domain whose architecture is characterized by a core of two parallel helices sandwiched between two other helices on either side. Despite an unusually high structural similarity contributed by the C-terminal helices and their preference for residues with high hydrophobicity across different species^S23^, much diversity is observed in the functional roles defined by this domain. These range from binding actin and the regulation of cytoskeleton to functioning as adaptors and regulators for signaling molecules in muscle and signal-transduction proteins^S24^. Although more structures in this area are wanting to explain the definitive functional roles of the multitude of members in this superfamily, diversity in function appears to be partly related to prominent subfamily specific differences observed in regions between the first two helices^S23^. The low sequence similarity between the different CH domain subfamilies could play an additional role^S25^. In addition, some of the protein members of this superfamily have a single copy of this domain while others adopt a tandem arrangement of multiple such domains (Tables S1 and S3). Here again, versatility in function is achieved through localized differences and involve minimal changes to the lengths of the core actin-binding domain.

**4) C2 domain (Calcium/lipid-binding domain)**

The C2 domains with a size of ~130 residues are independently folding protein modules, which can bind Ca^2+^ and perform important functional roles in signal transduction and membrane traffic. Ca^2+^ binding has been found to regulate binding of this domain with other interacting partners such as phospholipids, syntaxin, inositol phosphates, *etc.,*^S26^. The domain structure is characterized by an 8-stranded β-sandwich scaffold that is connected at the top and bottom by three and four loops respectively. Ca^2+^ binding occurs exclusively in the top three loops. A high degree of structural similarity occurs in the core β-sandwich while much diversity in sequence is observed in the top and bottom loops thus permitting the emergence of functional specificity^S27^.

**5) Nucleoside phosphorylase**

Two folds have evolved independently in nature to undertake the reversible phosphorolysis of purine and pyrimidine nucleosides. The NP1 family is one such, with an average domain size of ~250 residues. The NP1 enzymes, share a common domain subunit, although specified by diverse sequences. Also, they employ different quaternary contexts as trimers or hexamers and accept a range of purine and pyrimidine substrates^S28^. Sequence comparisons of the proteins belonging to this superfamily suggest that sequences likely to function as trimers cluster distinctly from those, which are hexamers. Interestingly, the differences in sequence composition largely dictate ultimate structural contexts and affect the differential recognition of diverse substrates. As in all length-rigid superfamilies, here also structural integrity is strictly maintained between members. All NP1 superfamily members conserve a central β-sheet structural motif in which the nucleoside and phosphate groups bind the C-terminal ends of the strands despite differences in the sequence of these regions. Additionally, conserved helices in all members of the superfamily play important structural roles in conferring stability to the fold.

A general theme that appears to emerge from the ‘length-rigid superfamilies’ that we have examined is that such proteins are less tolerant to modifications in the lengths of their domain. This conservative nature, however, does not appear to restrain the evolution of functional diversity and in the examples studied, we find that sequence composition and lengths of some of the loops defining the active site can undergo minor modifications. In some cases, structural contexts are dictated by sequence composition. Interestingly, such superfamilies are not as well-populated as the ‘length-deviant superfamilies’ which are often more promiscuous in their function. On the other hand, for some superfamilies, functional constraints appear to restrict the extent of length variation and these have been discussed in more detail elsewhere^S29^. These include superfamilies such as the cytochrome P450, lipocalins and many proteins involved in cell-signaling where the maintenance of the core scaffold is vital for function and drastic alterations are avoided since this can affect interaction interfaces. Thus, changes in the orientation of structures, modifying the sequences specific to the active site and introducing short structures to modify the active site locally are some of the strategies employed by ‘length-rigid’ proteins to attain functional diversity while still retaining structural integrity.

**References**

S1. Cunane, L.M., Chen, Z.W., Shamala, N., Mathews, F.S., Cronin, C.N. & McIntire, W.S. (2000). Structures of the flavocytochrome p-cresol methylhydroxylase and its enzyme-substrate complex: gated substrate entry and proton relays support the proposed catalytic mechanism. *J Mol Biol* 295, 357-374.

S2. Than, M.E., Hof, P., Huber, R., Bourenkov, G.P., Bartunik, H.D., Buse, G. & Soulimane, T. (1997). Thermus thermophilus cytochrome-c552: A new highly thermostable cytochrome-c structure obtained by MAD phasing. *J Mol Biol* 271, 629-644.

S3. Benson, S.D., Bamford, J.K., Bamford, D.H. & Burnett, R.M. (1999). Viral evolution revealed by bacteriophage PRD1 and human adenovirus coat protein structures. *Cell* 98, 825-833.

S4. Rux, J.J., Kuser, P.R. & Burnett, R.M. (2003). Structural and phylogenetic analysis of adenovirus hexons by use of high-resolution x-ray crystallographic, molecular modeling, and sequence-based methods. *J Virol* 77, 9553-9566.

S5. Leiros, I., Secundo, F., Zambonelli, C., Servi, S. & Hough, E. (2000). The first crystal structure of a phospholipase D. *Structure* 8, 655-667.

S6. Stuckey, J.A. & Dixon, J.E. (1999). Crystal structure of a phospholipase D family member. *Nat Struct Biol* 6, 278-284.

S7. Quiocho, F.A., Hu, G. & Gershon, P.D. (2000). Structural basis of mRNA cap recognition by proteins. *Curr Opin Struct Biol* 10, 78-86.

S8. Zhang, X., Zhou, L. & Cheng, X. (2000). Crystal structure of the conserved core of protein arginine methyltransferase PRMT3. *Embo J* 19, 3509-3519.

S9. Jain, D., Nair, D.T. Swaminathan, G.J., Abraham, E.G., Nagaraju, J., & Salunke, D.M. (2001). Structure of the induced antibacterial protein from tasar silkworm, Antheraea mylitta. Implications to molecular evolution. *J Biol Chem* 276, 41377-41382.

S10. van Asselt, E.J., Dijkstra, A.J., Kalk, K.H., Takacs, B., Keck, W. & Dijkstra, B.W. (1999). Crystal structure of Escherichia coli lytic transglycosylase Slt35 reveals a lysozyme-like catalytic domain with an EF-hand. *Structure* 7, 1167-1180.

S11. Campbell, R.E., Mosimann, S.C., van De Rijn, I., Tanner, M.E. & Strynadka, N.C. (2000). The first structure of UDP-glucose dehydrogenase reveals the catalytic residues necessary for the two-fold oxidation. *Biochemistry* 39, 7012-7023.

S12. Phillips, C., Dohnalek, J., Gover, S., Barrett, M.P. & Adams, M.J. (1998). A 2.8 A resolution structure of 6-phosphogluconate dehydrogenase from the protozoan parasite Trypanosoma brucei: comparison with the sheep enzyme accounts for differences in activity with coenzyme and substrate analogues. *J Mol Biol* 282, 667-681.

S13. Sinha, S.C. & Smith, J.L. (2001). The PRT protein family. *Curr Opin Struct Biol* 11, 733-739.

S14. Kabsch, W. & Holmes, K.C. (1995). The actin fold. *Faseb J* 9, 167-174.

S15. Buss, K.A., Cooper, D.R., Ingram-Smith, C., Ferry, J.G., Sanders, D.A. & Hasson, M.S. (2001). Urkinase: structure of acetate kinase, a member of the ASKHA superfamily of phosphotransferases. *J Bacteriol* 183, 680-686.

S16. Dunwell, J.M., Culham, A., Carter, C.E., Sosa-Aguirre, C.R. & Goodenough, P.W. (2001). Evolution of functional diversity in the cupin superfamily. *Trends Biochem Sci* 26, 740-746.

S17. Thayer, M.M., Ahern, H., Xing, D., Cunningham, R.P. & Tainer, J.A. (1995). Novel DNA binding motifs in the DNA repair enzyme endonuclease III crystal structure. *Embo J* 14, 4108-4120.

S18. Guan, Y., Manuel, R.C., Arvai, A.S., Parikh, S.S., Mol, C.D., Miller, J.H., Lloyd, S. & Tainer, J.A. (1998). MutY catalytic core, mutant and bound adenine structures define specificity for DNA repair enzyme superfamily. *Nat Struct Biol* 5, 1058-1064.

S19. Labahn, J., Scharer, O.D., Long, A., Ezaz-Nikpay, K., Verdine, G.L. & Ellenberger, T.E. (1996). Structural basis for the excision repair of alkylation-damaged DNA. *Cell* 86, 321-329.

S20. Todd, A.E., Orengo, C.A. & Thornton, J.M. (1999). Evolution of protein function, from a structural perspective. *Curr Opin Chem Biol* 3, 548-556.

S21. Bhaduri, A., Pugalenthi, G. & Sowdhamini, R. (2004). PASS2: an automated database of protein alignments organised as structural superfamilies. *BMC Bioinformatics* 5, 35.

S22. Baggiolini, M., Dewald, B. & Moser, B. (1997). Human chemokines: an update. *Annu Rev Immunol* 15, 675-705.

S23. Gimona, M., Djinovic-Carugo, K., Kranewitter, W.J. & Winder, S.J. (2002). Functional plasticity of CH domains. *FEBS Lett* 513, 98-106.

S24. Banuelos, S., Saraste, M. & Carugo, K.D. (1998). Structural comparisons of calponin homology domains: implications for actin binding. *Structure* 6, 1419-1431.

S25. Stradal, T., Kranewitter, W., Winder, S.J. & Gimona, M. (1998). CH domains revisited. *FEBS Lett* 431, 134-137.

S26. Nalefski, E.A., Wisner, M.A., Chen, J.Z., Sprang, S.R., Fukuda, M., Mikoshiba, K. & Falke, J.J. (2001). C2 domains from different Ca2+ signaling pathways display functional and mechanistic diversity. *Biochemistry* 40, 3089-3100.

S27. Rizo, J. & Sudhof, T.C. (1998). C2-domains, structure and function of a universal Ca2+-binding domain. *J Biol Chem* 273, 15879-15882.

S28. Pugmire, M.J. & Ealick, S.E. (2002). Structural analyses reveal two distinct families of nucleoside phosphorylases. *Biochem J* 361, 1-25.

S29. Reeves, G.A., Dallman, T.J., Redfern, O.C., Akpor, A. & Orengo, C.A. (2006). Structural diversity of domain superfamilies in the CATH database. *J Mol Biol* 360, 725-741.

S30. Von Mering, C., Jensen, L. J., Kuhn, M., Chaffron, S., Doerks, T., Kruger, B., Snel, B. & Bork, P. (2007). STRING 7-recent developments in the integration and prediction of protein interactions. *Nucleic Acids Res* 35, D358-62.

**Figure legend:**

1. S1: The two-domain scaffold of the DNA-glycosylase domain superfamily in Adenine glycosylase and Endonuclease III harbors a HhH motif (in pink) with active site residues (in red) to bind their respective substrates. Composition of residues in the active site is distinct for each member.
2. S2: Interleukin-8 like chemokine superfamily shows high conservation of the core structure. Lymphotactin (a) and stromal cell-derived factor 1 alpha (b) differ primarily in the N- and C-termini. Lower panel (c) shows a graphical projection of the alignments. The core structure involving the well conserved 3_10_ helix and the three-stranded sheet is well conserved across different members and structurally equivalent regions in the alignment are extensive.(Helix- red, strand – blue, coil – green, indels- magenta).

**Supplementary table legend:**

**Table S1) Structural repeats in length-deviant and length-rigid protein domain**

**superfamilies**

**Table S2) Domain contexts of all length-deviant domain superfamilies**. The number of occurrences of domains singly, multiply and as repeats (tandem repeats of domain) in a single or multiple chain are provided.

**Table S3) Domain contexts in length-rigid domain superfamilies**. The number of occurrences of domains in single (SD), multiple (MD) and as R (tandem repeats of domain) in a single or multiple chain are provided. R+MD includes domain occurrences that show repeating copies of self-domain in multi-domain contexts.

**Table S4)** **Number of protein-protein interactions (known and predicted) in length-deviant and length-rigid domain superfamilies after searching in STRING database^S30^**.

**Table S5) Structural and functional role of indels in the 64 length-deviant domain superfamilies.**
