# Supplementary figures and images for: Length Variations amongst Protein Domain Superfamilies and Consequences on Structure and Function
Source: PLoS One. 2009 Mar 31;4(3):e4981. doi: 10.1371/journal.pone.0004981 (PMC2659687; doi:10.1371/journal.pone.0004981)

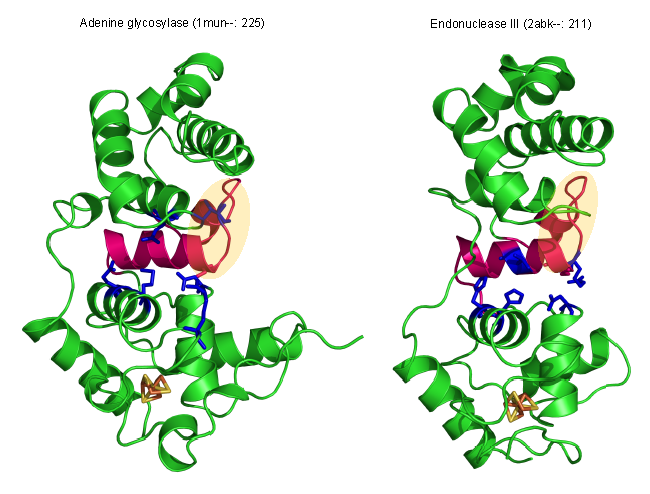

Supplement: Figure S1 — DNA-glycosylase domain superfamily. The two domain scaffold of the DNA-glycosylase domain superfamily in Adenine glycosylase and Endonuclease III harbors a HhH motif (in pink) with active site residues (in red) to bind their respective substrates. Composition of residues in the active site is distinct for each member. (0.51 MB TIF) [file pone.0004981.s002.tif]

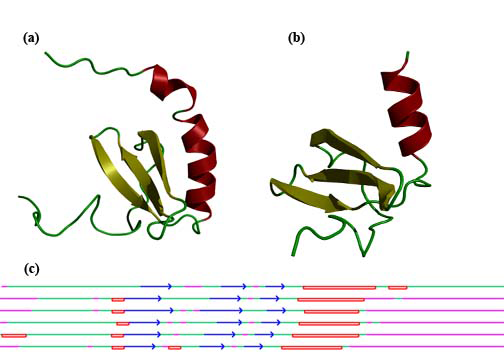

Supplement: Figure S2 — Interleukin-8-like superfamily. Interleukin-8 like chemokine superfamily shows high conservation of the core structure. Lymphotactin (a) and stromal cell derived factor 1 alpha (b) differ primarily in the N and C termini. Lower panel (c) shows a graphical projection of the alignments. The core structure involving the well conserved 310 helix and the three stranded sheet is well conserved across different members and structurally equivalent regions in the alignment are extensive.(Helix- red, strand - blue, coil - green, indels- magenta) (0.15 MB TIF) [file pone.0004981.s003.tif]
