# Supplementary material for: Length Variations amongst Protein Domain Superfamilies and Consequences on Structure and Function
Source: PLoS One. 2009 Mar 31;4(3):e4981. doi: 10.1371/journal.pone.0004981 (PMC2659687; doi:10.1371/journal.pone.0004981)
Supplement: Table S1 — Structural repeats in length-deviant and length-rigid protein domain superfamilies (1.27 MB DOC) [file pone.0004981.s004.doc]

**Table S1: Structural repeats in length-deviant and length- rigid protein domain superfamilies**

| **No** | **Superfamily** | | **Av_**  **Domain size** | **No_mem** | **Member with domain repeat** | **Structural match**  **(residues)** | **Match score (RMSD)** | **Superposition of repeat domains** |
| --- | --- | --- | --- | --- | --- | --- | --- | --- |
| **Length-deviant domain superfamilies** | | |  |  |  |  |  |  |
| 1 | | Cytochrome C | 101 | 22 | 1iqc:  1iqca1:150  1iqca2:158 | 85 | 1.141 | 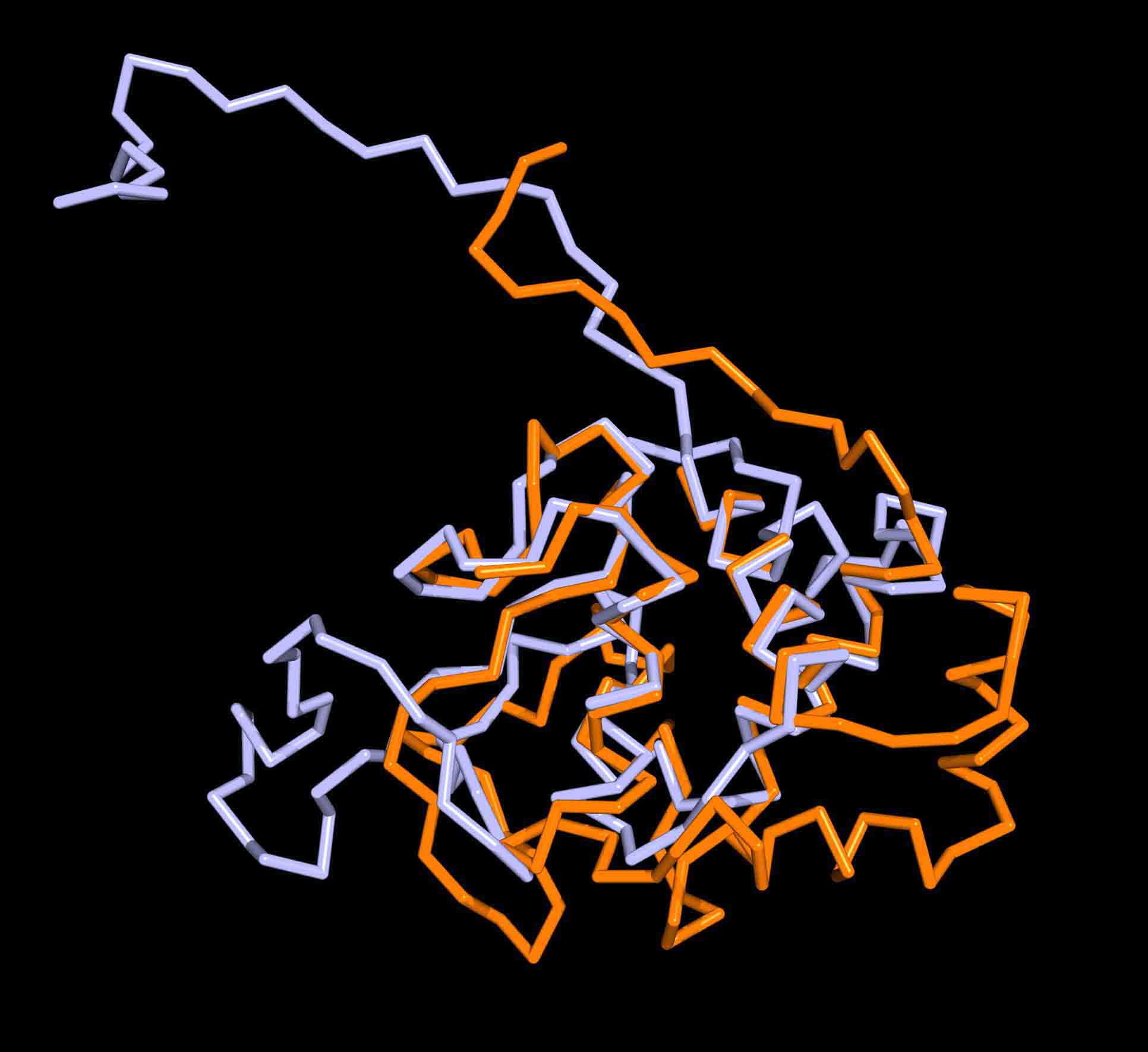 |
|  | |  |  |  |  |  |  |  |
| 2 | | Viral proteins | 313 | 4 | 1hx6:  1hx6a2:140  1hx6a1:230 | 102 | 1.790 | 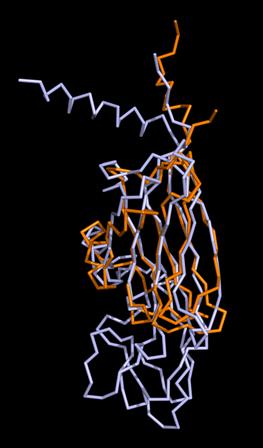 |
|  | |  |  |  |  |  |  |  |
| 3 | | RmlC-like Cupins* | 243 | 8 | 1fxz:  1fxza2:174  1fxza1:197 | 146 | 1.200 | 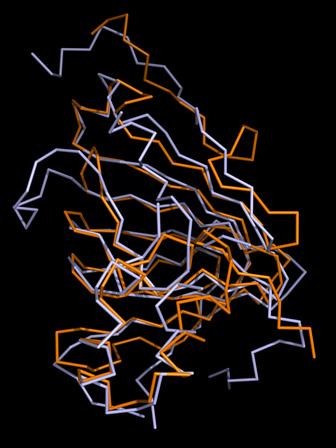 |
|  | |  |  |  |  |  |  |  |
| 4 | | Actin_like ATPase domain | 205 | 7 | 1bu6:  1bu6o1:251  1bu6o2:246 | 70 | 1.638 | 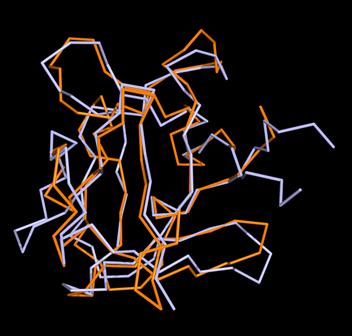 |
|  | |  |  |  |  |  |  |  |
| 5 | | PRTase like | 194 | 14 | 1dkr:  1dkra2:141  1dkra1:157 | 105 | 1.562 | 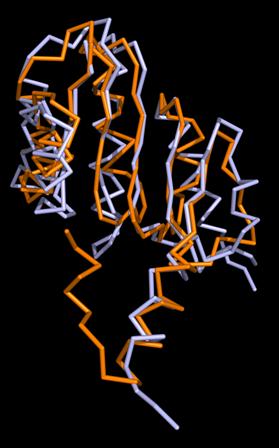 |
|  | |  |  |  |  |  |  |  |
| 6 | | Phospholipase D | 215 | 5 | 1f0i:  1f0ia1:256  1f0ia2:240 | 138 | 1.615 | 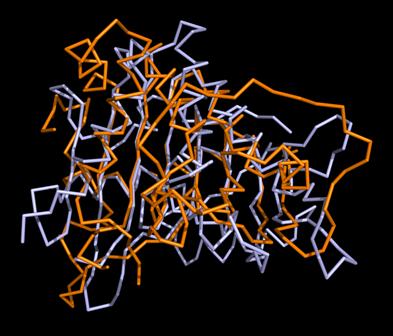 |
|  | |  |  |  |  |  |  |  |
| 7 | | Concanavalin A like lectin* | 197 | 26 | 1dyk  1dyka1:188  1dyka2:184 | 157 | 1.240 | 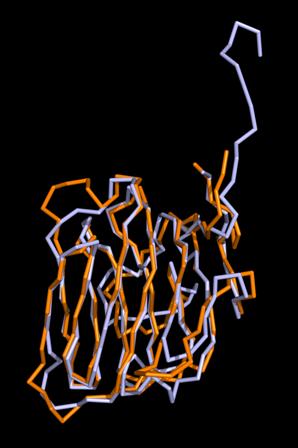 |
|  | |  |  |  |  |  |  |  |
| 8 | | Nucleic acid_binding proteins | 112 | 39 | 1jb7:  1jb7a1:169  1jb7a2:124 | 80 | 1.486 | 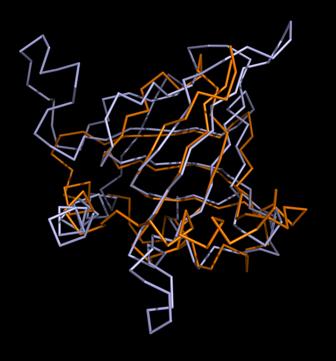 |
|  | |  |  |  |  |  |  |  |
| 9 | | E_set domains | 122 | 42 | 1g4m:  1g4ma1:171  1g4ma2:185 | 94 | 1.506 | 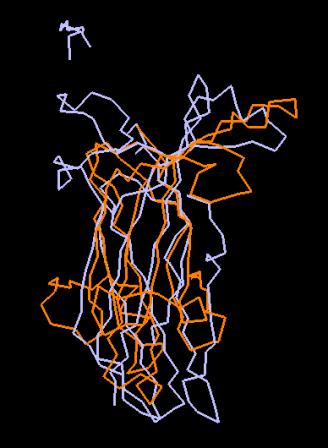 |
|  | |  |  |  |  |  |  |  |
| 10 | | Thio-redoxin like | 109 | 42 | 1a8l:  1a8la1:124  1a8la2:112 | 77 | 1.866 | 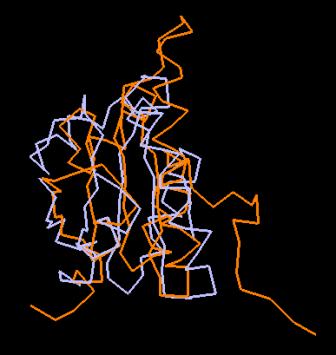 |
|  | |  |  |  |  |  |  |  |
| 11 | | Protein tyrosine Phosphatase II* | 234 | 12 | 1lar:  1lara1:317  1lar2:249 | 242 | 0.999 | 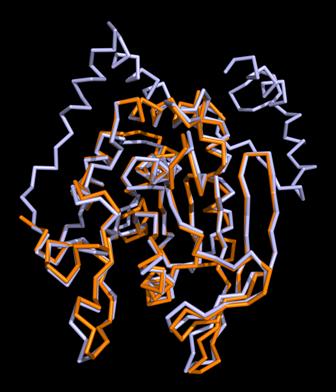 |
|  | |  |  |  |  |  |  |  |
| 12 | | P-loop containing NTP hydrolase | 221 | 63 | 1a1v:  1a1va1:135  1a1va2:294 | 84 | 1.634 | 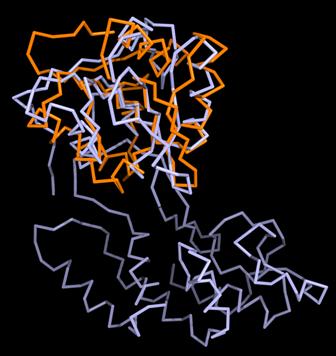 |
|  | |  |  |  |  |  |  |  |
| 13 | | Helical backbone metal receptor | 400 | 7 | 1mio:  1mio:525  1mio:457 | 280 | 1.877 | 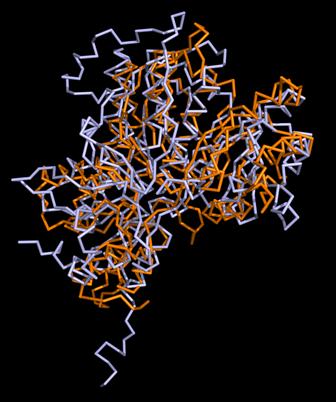 |
|  | |  |  |  |  |  |  |  |
| 14 | | Periplasmic binding protein-like II* | 255 | 15 | 1cbb:  1cbba:357  1cbba:334 | 163 | 1.087 | 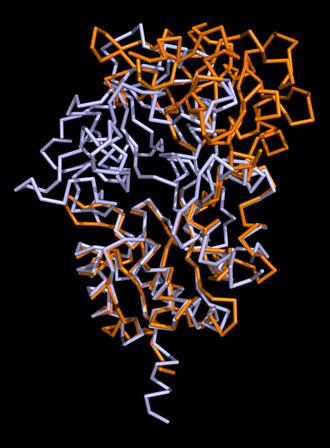 |
|  | |  |  |  |  |  |  |  |
| 15 | | Thiolase like | 193 | 12 | 1afw:  1afwa1:266  1afwa2:124 | 92 | 1.905 | 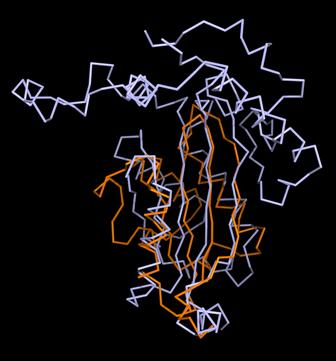 |
|  | |  |  |  |  |  |  |  |
| 16 | | MHC antigen recognition domain | 143 | 13 | 1hdm:  1hdma2:81  1hdma1:85 | 57 | 1.497 | 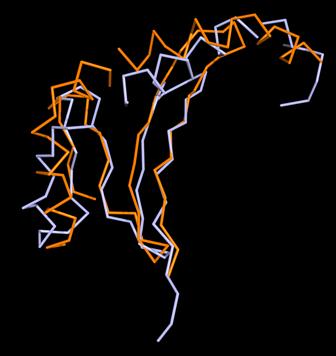 |
|  | |  |  |  |  |  |  |  |
| 17 | | Tetrahydrobiopterin biosynthesis enzyme like | 155 | 7 | 1uox:  1uox1:136  1uox2:157 | 102 | 1.221 | 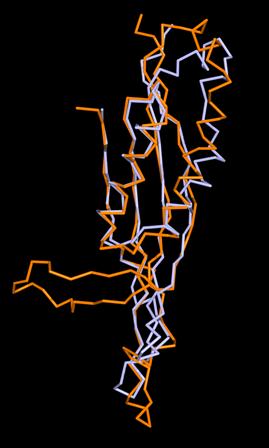 |
|  | |  |  |  |  |  |  |  |
| 18 | | AcylCoA-N acyltransferase | 194 | 10 | 1iic:  1iica:185  1iica:237 | 101 | 1.620 | 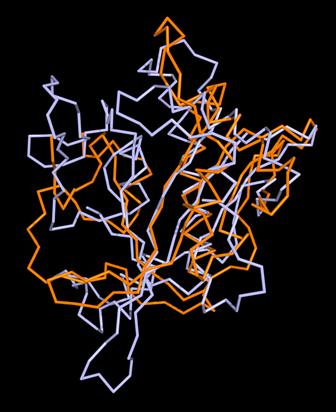 |
|  | |  |  |  |  |  |  |  |
| 19 | | Ferritin like* | 259 | 12 | 1mty:  1mtyd:512  1mtyb:384 | 226 | 1.827 | 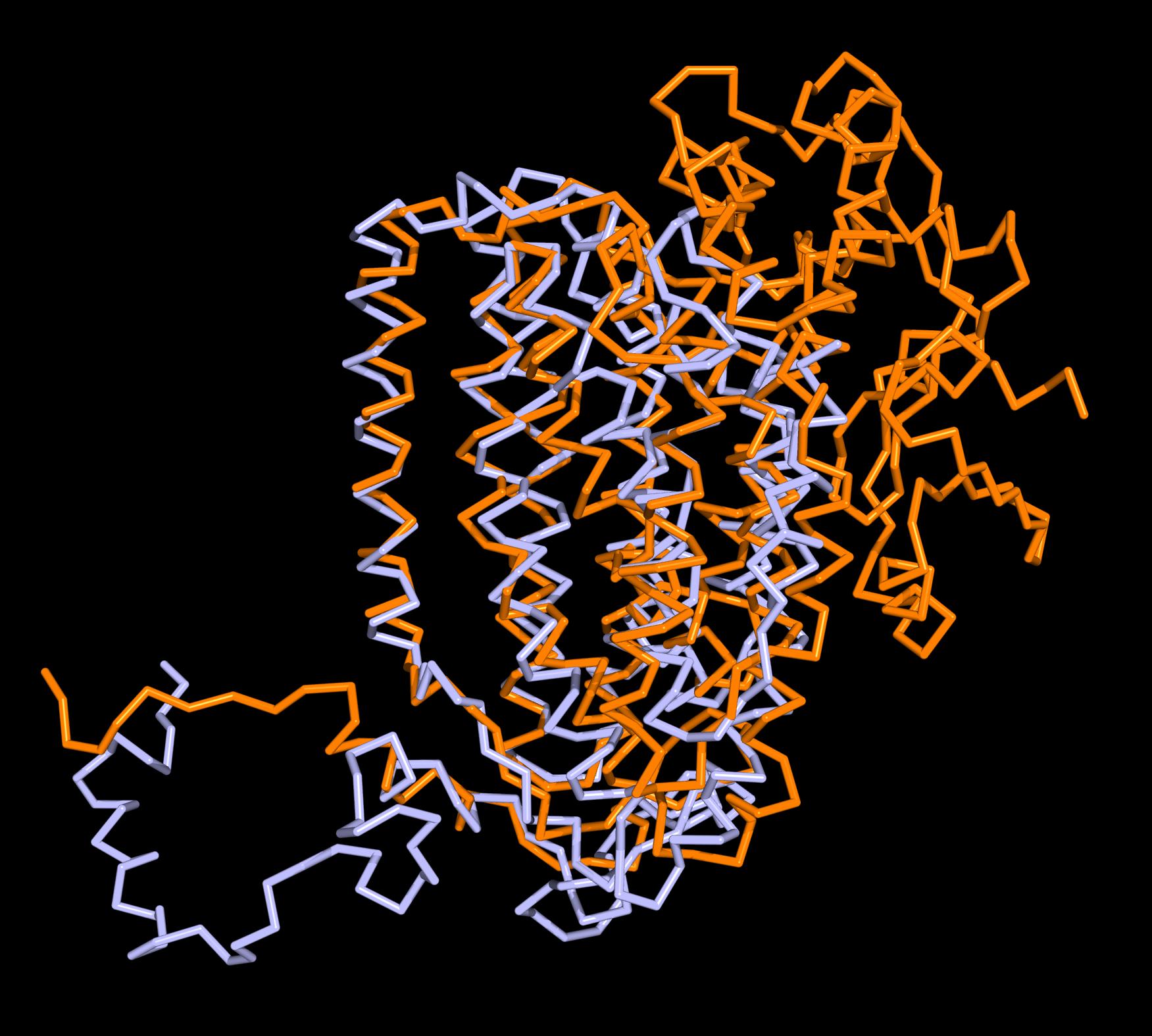 |
|  | |  |  |  |  |  |  |  |
| 20 | | EF-hand* | 125 | 35 | 1eg3:  1eg3a1:125  1eg3a2:97 | 47 | 1.609 | 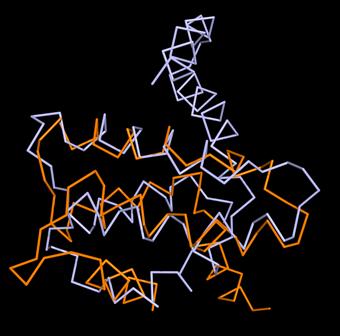 |
|  | |  |  |  |  |  |  |  |
| 21 | | Terpenoid cyclase* | 308 | 6 | 2sqc:  2sqca1:352  2sqca2:271 | 97 | 1.514 | 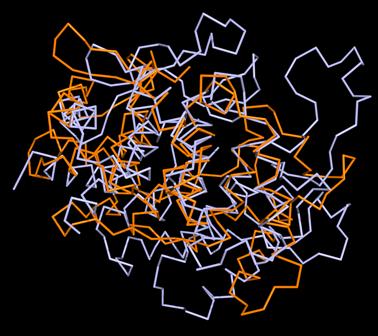 |
|  | |  |  |  |  |  |  |  |
| 22 | | Cupredoxin | 146 | 32 | 1aoz:  1aoza3:214  1aoza2:209 | 104 | 1.415 | 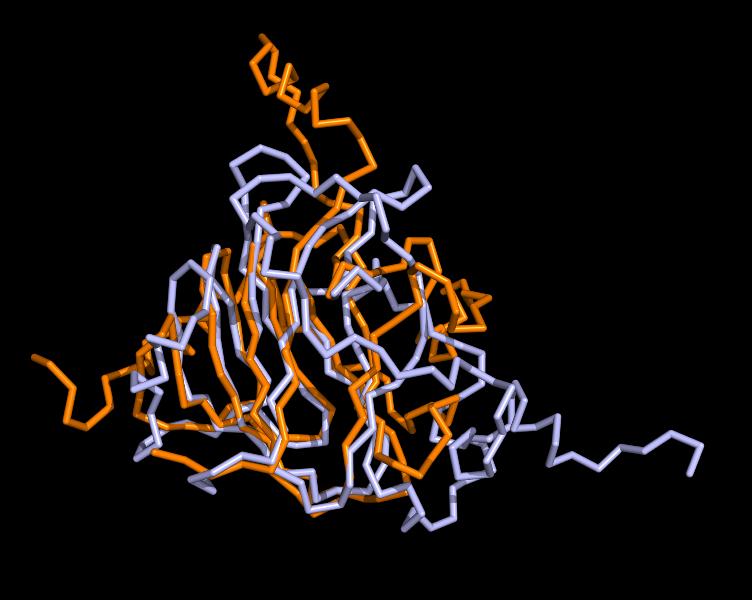 |
|  | |  |  |  |  |  |  |  |
| 23 | | Viral coat and capsid proteins | 227 | 31 | 1a6c:  1a6ca1:176  1a6ca2:172 | 83 | 1.652 | 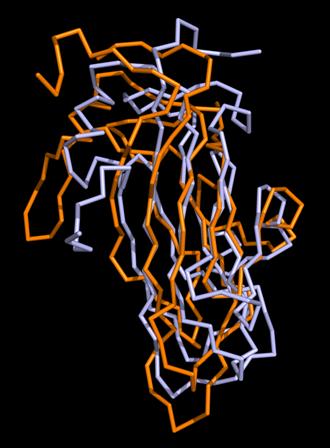 |
|  | |  |  |  |  |  |  |  |
| 24 | | Homeodomain like | 64 | 32 | 1ign:  1igna1:103  1igna2:86 | 52 | 1.065 | 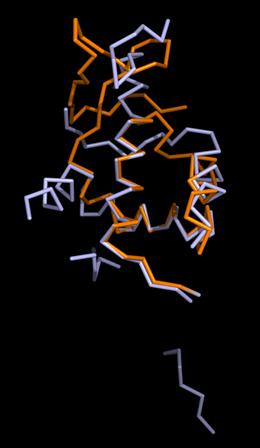 |
|  | |  |  |  |  |  |  |  |
| 25 | | Putative DNA binding domain* | 90 | 5 | 1jjc:  1jjcb1:77  1jjcb2:75 | 60 | 1.743 | 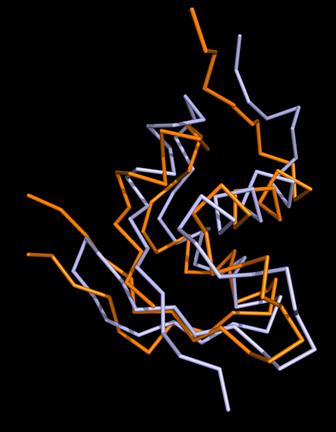 |
|  | |  |  |  |  |  |  |  |
| 26 | | Histone fold | 88 | 12 | 1bh9:  1bh9a:45  1bh9b:89 | 42 | 0.872 | 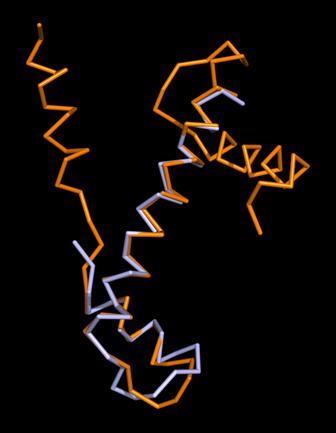 |
|  | |  |  |  |  |  |  |  |
| 27 | | Winged helix DNA binding domain* | 88 | 48 | 2fok:  2foka:138  2foka3:95 | 60 | 1.491 | 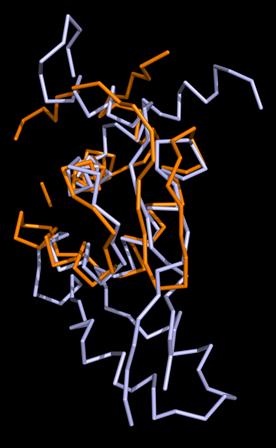 |
| **Length-rigid domain superfamilies** | | |  |  |  |  |  |  |
| 28 | | Calponin homology domain,CHD | 114 | 5 | 1aoa:  1a0a1:131  1aoa2:116 | 86 | 1.491 | 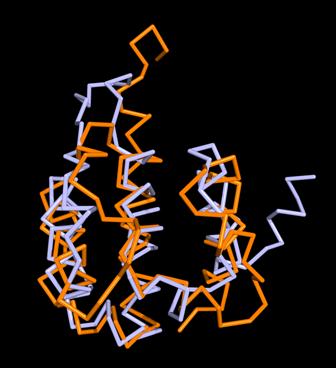 |
|  | |  |  |  |  |  |  |  |
| 29 | | cAMP binding domain like | 135 | 5 | 1cx4:  1cx4a1:136  1cx4a2:139 | 108 | 1.205 | 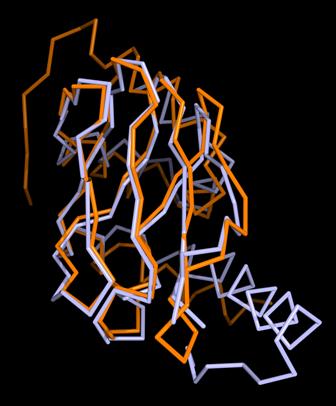 |
|  | |  |  |  |  |  |  |  |
| 30 | | Actin crosslinking proteins | 118 | 5 | 1dfc:  1dfca1:125  1dfca2:119 | 104 | 1.276 | 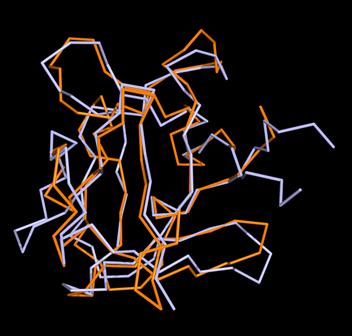 |
|  | |  |  |  |  |  |  |  |
| 31 | | Invasin/Intimin cell adhesion fragments | 94 | 6 | 1cwv:  1cwva1:103  1cwva2:96 | 89 | 0.919 | 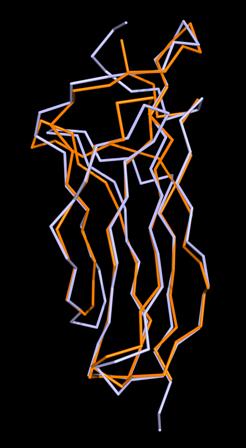 |
|  | |  |  |  |  |  |  |  |
| 32 | | Sm-like ribonucleoproteins | 75 | 5 | 1d3b:  1d3ba:72  1d3bb:81 | 64 | 1.151 | 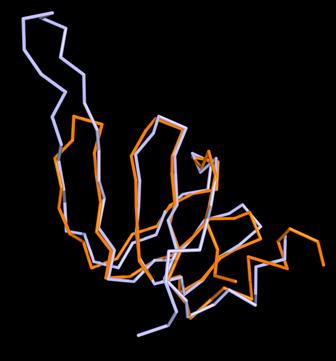 |
|  | |  |  |  |  |  |  |  |
| 33 | | UBC-like | 151 | 7 | 1jat:  1jatb:152  1jata:132 | 109 | 1.302 | 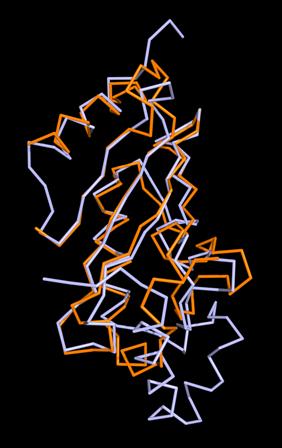 |
|  | |  |  |  |  |  |  |  |
| 34 | | DNA clamp | 124 | 12 | 1dml:  1dmla1:141  1dmla2:126 | 69 | 1.674 | 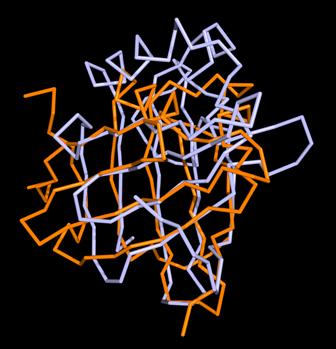 |
|  | |  |  |  |  |  |  |  |
| 35 | | RNA binding domain | 87 | 17 | 1cvj:  1cvja1:80  1cvja2:89 | 76 | 1.118 | 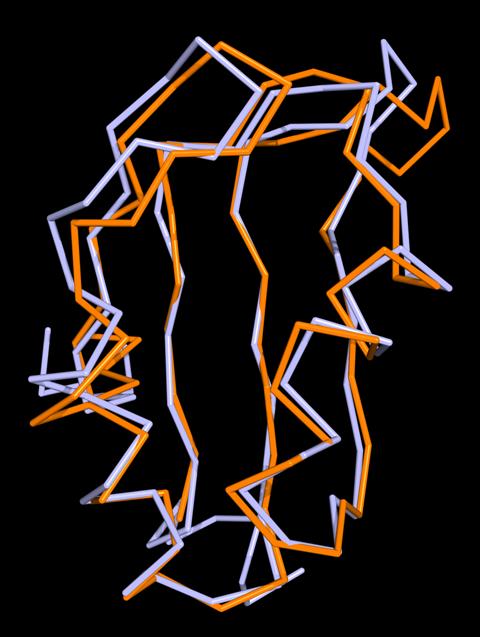 |

**Abbreviations employed in the table:**

Av_dom: Average domain size of the superfamily

No_mem: Number of members

Match score: RMSD between matched residues (between the members compared)

*: Repeats also detected through submissions in TRUST server.
