# Supplementary material for: Length Variations amongst Protein Domain Superfamilies and Consequences on Structure and Function
Source: PLoS One. 2009 Mar 31;4(3):e4981. doi: 10.1371/journal.pone.0004981 (PMC2659687; doi:10.1371/journal.pone.0004981)
Supplement: Table S2 — The number of occurrences of domains singly, multiply and as repeats (tandem repeats of domain) in a single or multiple chain are provided. (0.31 MB DOC) [file pone.0004981.s005.doc]

**Table S2:** Domain contexts of all length-deviant domain superfamilies. The numbers of occurrences of domains singly, multiply and as repeats (tandem repeats of domain) in a single or multiple chain are provided.

|  |  | **Single chain** | | | **Multiple chains** | | |  |  |  |
| --- | --- | --- | --- | --- | --- | --- | --- | --- | --- | --- |
| **S.No** | **Superfamily** | **Single Domain** | **Multi Domain** | **Repeat** | **Single**  **Domain** | **Multi Domain** | **Repeat** | **Total** | **Domain Repeats** | **Oligomers** |
|  | **Concanavalin A like lectins/glucanase** |  |  |  |  |  |  |  |  |  |
| **1** |  | **15** | **6** | **4** | **12** | **3** | **-** | **40** | **Y** | **Y** |
|  |  |  |  |  |  |  |  |  |  |  |
| **2** | **SH3-domain** | **19** | **3** | **1** | **7** | **3** | **-** | **33** | **Y** | **Y** |
|  |  |  |  |  |  |  |  |  |  |  |
| **3** | **Translation proteins SH3-like domain** | **-** | **2** | **-** | **-** | **4** | **-** | **6** | **N** | **Y** |
|  |  |  |  |  |  |  |  |  |  |  |
| **4** | **GroES-like** | **3** | **1** | **-** | **5** | **3** | **-** | **12** | **N** | **Y** |
|  |  |  |  |  |  |  |  |  |  |  |
| **5** | **PDZ domain-like** | **29** | **1** | **1** | **5** | **3** | **2** | **41** | **Y** | **Y** |
|  |  |  |  |  |  |  |  |  |  |  |
| **6** | **Bacterial enterotoxins** | **-** | **5** | **-** | **2** | **10** | **-** | **17** | **N** | **Y** |
|  |  |  |  |  |  |  |  |  |  |  |
| **7** | **Nucleic acid-binding proteins** | **6** | **7** | **-** | **5** | **13** | **2** | **33** | **Y** | **Y** |
|  |  |  |  |  |  |  |  |  |  |  |
| **8** | **Trypsin-like serine proteases** | **9** | **1** | **-** | **18** | **1** | **1** | **30** | **Y** | **Y** |
|  |  |  |  |  |  |  |  |  |  |  |
| **9** | **ADC-like** | **-** | **3** | **-** | **3** | **3** | **-** | **9** | **N** | **Y** |
|  |  |  |  |  |  |  |  |  |  |  |
| **10** | **PK beta-barrel domain-like** | **-** | **-** | **-** | **-** | **3** | **-** | **3** | **N** | **Y** |
|  |  |  |  |  |  |  |  |  |  |  |
| **11** | **alpha-Amylases, C-terminal beta-sheet domain** | **-** | **14** | **-** | **-** | **13** | **-** | **27** | **N** | **Y** |
|  |  |  |  |  |  |  |  |  |  |  |
| **12** | **WW domain** | **4** | **2** | **2** | **-** | **-** | **-** | **8** | **Y** | **N** |
|  |  |  |  |  |  |  |  |  |  |  |
| **13** | **Rudiment single hybrid motif** | **-** | **1** | **-** | **-** | **5** | **-** | **6** | **N** | **Y** |
|  |  |  |  |  |  |  |  |  |  |  |
| **14** | **E set domains** | **3** | **16** | **-** | **5** | **14** | **4** | **42** | **Y** | **Y** |
|  |  |  |  |  |  |  |  |  |  |  |
| **15** | **(Trans)glycosidases** | **19** | **15** | **-** | **7** | **5** | **-** | **46** | **N** | **Y** |
|  |  |  |  |  |  |  |  |  |  |  |
| **16** | **Phosphoenolpyruvate/pyruvate domain** | **1** | **1** | **-** | **6** | **1** | **-** | **9** | **N** | **Y** |
|  |  |  |  |  |  |  |  |  |  |  |
| **17** | **Adenine nucleotide alpha hydrolases-like** | **1** | **2** | **-** | **2** | **4** | **-** | **9** | **N** | **Y** |
|  |  |  |  |  |  |  |  |  |  |  |
| **18** | **Thioredoxin-like** | **14** | **7** | **-** | **7** | **10** | **1** | **39** | **Y** | **Y** |
|  |  |  |  |  |  |  |  |  |  |  |
| **19** | **P-loop containing nucleotide triphosphate hydrolases** | **18** | **16** | **4** | **4** | **11** | **2** | **55** | **Y** | **Y** |
|  |  |  |  |  |  |  |  |  |  |  |
| **20** | **(Phosphotyrosine protein) phosphatases II** | **3** | **1** | **-** | **4** | **2** | **-** | **10** | **Y** | **Y** |
|  |  |  |  |  |  |  |  |  |  |  |
| **21** | **Aminoacid dehydrogenase-like, N-terminal domain** | **-** | **2** | **-** | **-** | **7** | **-** | **9** | **Y** | **Y** |
|  |  |  |  |  |  |  |  |  |  |  |
| **22** | **S-adenosyl-L-methionine-dependent methyltransferases** | **25** | **3** | **-** | **15** | **3** | **-** | **46** | **N** | **Y** |
|  |  |  |  |  |  |  |  |  |  |  |
| **23** | **Nucleotide-diphospho-sugar transferases** | **6** | **1** | **-** | **9** | **1** | **-** | **17** | **N** | **Y** |
|  |  |  |  |  |  |  |  |  |  |  |
| **24** | **alpha/beta-Hydrolases** | **20** | **3** | **-** | **15** | **1** | **-** | **39** | **N** | **Y** |
|  |  |  |  |  |  |  |  |  |  |  |
| **25** | **"Helical backbone" metal receptor** | **2** | **-** | **-** | **6** | **-** | **-** | **8** | **N** | **Y** |
|  |  |  |  |  |  |  |  |  |  |  |
| **26** | **Periplasmic binding protein-like I** | **3** | **1** | **-** | **8** | **-** | **-** | **12** | **N** | **Y** |
|  |  |  |  |  |  |  |  |  |  |  |
| **27** | **Periplasmic binding protein-like II** | **8** | **1** | **2** | **4** | **-** | **-** | **15** | **Y** | **Y** |
|  |  |  |  |  |  |  |  |  |  |  |
| **28** | **Thiolase-like** | **-** | **-** | **-** | **-** | **-** | **8** | **8** | **N** | **Y** |
|  |  |  |  |  |  |  |  |  |  |  |
| **29** | **Ankyrin repeat** | **2** | **-** | **-** | **2** | **2** | **-** | **6** | **Y** | **Y** |
|  |  |  |  |  |  |  |  |  |  |  |
| **30** | **Cysteine proteinases** | **2** | **-** | **-** | **5** |  | **-** | **7** | **N** | **Y** |
|  |  |  |  |  |  |  |  |  |  |  |
| **31** | **Ribosomal protein S5 domain 2-like** | **2** | **3** | **1** |  | **2** | **-** | **8** | **Y** | **Y** |
|  |  |  |  |  |  |  |  |  |  |  |
| **32** | **FAD-linked reductases, C-terminal domain** | **-** | **2** | **-** | **-** | **9** | **-** | **11** | **N** | **Y** |
|  |  |  |  |  |  |  |  |  |  |  |
| **33** | **MHC antigen-recognition domain** | **-** | **-** | **-** | **2** | **9** | **-** | **11** | **N** | **Y** |
|  |  |  |  |  |  |  |  |  |  |  |
| **34** | **POZ domain** | **4** | **-** | **-** | **-** | **6** | **-** | **10** | **N** | **Y** |
|  |  |  |  |  |  |  |  |  |  |  |
| **35** | **4Fe-4S ferredoxins** | **6** | **1** | **-** | **1** | **2** | **-** | **10** | **N** | **Y** |
|  |  |  |  |  |  |  |  |  |  |  |
| **36** | **Tetrahydrobiopterin biosynthesis enzymes-like** | **1** | **-** | **1** | **4** | **-** | **-** | **6** | **Y** | **Y** |
|  |  |  |  |  |  |  |  |  |  |  |
| **37** | **C-type lectin-like** | **12** | **4** | **-** | **8** | **3** | **1** | **28** | **Y** | **Y** |
|  |  |  |  |  |  |  |  |  |  |  |
| **38** | **Acyl-CoA N-acyltransferases (Nat)** | **12** | **-** | **2** | **15** | **-** | **1** | **30** | **Y** | **Y** |
|  |  |  |  |  |  |  |  |  |  |  |
| **39** | **Ferritin like** | **3** | **2** | **-** | **10** | **2** | **-** | **17** | **N** | **Y** |
|  |  |  |  |  |  |  |  |  |  |  |
| **40** | **4 helical cytokines** | **8** | **-** | **-** | **13** | **1** | **-** | **22** | **N** | **Y** |
|  |  |  |  |  |  |  |  |  |  |  |
| **41** | **EF hand** | **15** | **-** | **1** | **14** | **4** | **-** | **34** | **Y** | **Y** |
|  |  |  |  |  |  |  |  |  |  |  |
| **42** | **IHF like DNA binding proteins** | **1** | **-** | **-** | **4** | **-** | **-** | **5** | **N** | **Y** |
|  |  |  |  |  |  |  |  |  |  |  |
| **43** | **Terpenoid cyclase, Protein prenyl transferase** | **1** | **1** | **1** | **-** | **1** | **1** | **5** | **Y** | **Y** |
|  |  |  |  |  |  |  |  |  |  |  |
| **44** | **ARM repeat** | **2** | **-** | **1** | **2** | **3** | **1** | **9** | **Y** | **Y** |
|  |  |  |  |  |  |  |  |  |  |  |
| **45** | **TPR like** | **5** | **1** | **-** | **3** | **-** | **-** | **9** | **N** | **Y** |
|  |  |  |  |  |  |  |  |  |  |  |
| **46** | **Carbohydrate binding domain** | **2** | **1** | **-** | **3** | **1** | **-** | **7** | **N** | **Y** |
|  |  |  |  |  |  |  |  |  |  |  |
| **47** | **P53 like transcription factor** | **1** | **2** | **-** |  | **3** | **-** | **6** | **N** | **Y** |
|  |  |  |  |  |  |  |  |  |  |  |
| **48** | **Cupredoxin** | **4** | **-** | **3** | **8** | **3** | **3** | **21** | **Y** | **Y** |
|  |  |  |  |  |  |  |  |  |  |  |
| **49** | **Viral coat and capsid proteins** | **1** | **-** | **1** | **4** | **-** | **-** | **6** | **Y** | **Y** |
|  |  |  |  |  |  |  |  |  |  |  |
| **50** | **Cytochrome c** | **17** | **4** | **-** | **5** | **1** | **6** | **33** | **Y** | **Y** |
|  |  |  |  |  |  |  |  |  |  |  |
| **51** | **6-Phosphogluconate dehydrogenase C-terminal like** | **1** | **5** | **-** | **5** | **1** | **1** | **13** | **Y** | **Y** |
|  |  |  |  |  |  |  |  |  |  |  |
| **52** | **Viral proteins** | **-** | **-** | **2** | **-** | **-** | **3** | **5** | **Y** | **Y** |
|  |  |  |  |  |  |  |  |  |  |  |
| **53** | **Rmlc-like Cupins** | **14** | **-** | **-** | **17** | **-** | **2** | **33** | **Y** | **Y** |
|  |  |  |  |  |  |  |  |  |  |  |
| **54** | **PRTase-like** | **2** | **-** | **5** | **8** | **1** | **3** | **19** | **Y** | **Y** |
|  |  |  |  |  |  |  |  |  |  |  |
| **55** | **Actin--like ATPase domain** | **1** | **-** | **7** | **1** | **-** | **10** | **19** | **Y** | **Y** |
|  |  |  |  |  |  |  |  |  |  |  |
| **56** | **Homeodomainlike** | **10** | **2** | **2** | **2** | **5** | **4** | **25** | **Y** | **Y** |
|  |  |  |  |  |  |  |  |  |  |  |
| **57** | **C-terminal effector domain of bipartite response regulator** | **4** | **-** | **-** | **3** | **1** | **-** | **8** | **N** | **Y** |
|  |  |  |  |  |  |  |  |  |  |  |
| **58** | **Putative DNA binding domain** | **5** | **2** | **-** | **4** | **-** | **1** | **12** | **Y** | **Y** |
|  |  |  |  |  |  |  |  |  |  |  |
| **59** | **Histone fold like** | **3** | **-** | **-** | **6** | **2** | **-** | **11** | **N** | **Y** |
|  |  |  |  |  |  |  |  |  |  |  |
| **60** | **Met repressor like** | **-** | **-** | **-** | **6** | **-** | **-** | **6** | **N** | **Y** |
|  |  |  |  |  |  |  |  |  |  |  |
| **61** | **Winged helix DNA binding domain** | **15** | **7** | **1** | **6** | **14** | **1** | **44** | **Y** | **Y** |
|  |  |  |  |  |  |  |  |  |  |  |
| **62** | **NADP binding**  **Rossman fold domain** | **2** | **16** | **-** | **2** | **28** | **-** | **48** | **N** | **Y** |
|  |  |  |  |  |  |  |  |  |  |  |
| **63** | **Phospholipase D** | **1** | **-** | **-** | **-** | **-** | **4** | **5** | **Y** | **Y** |
|  |  |  |  |  |  |  |  |  |  |  |
| **64** | **Lysozyme-like** | **6** | **-** | **-** | **3** | **1** | **1** | **11** | **Y** | **Y** |
|  | **Total** | **373** | **168** | **42** | **315** | **228** | **63** | **1189** |  |  |
|  | **Occurrence(%)** | **31.4** | **14.1** | **3.5** | **26.5** | **19.2** | **5.3** |  |  |  |
