# Supplementary material for: Length Variations amongst Protein Domain Superfamilies and Consequences on Structure and Function
Source: PLoS One. 2009 Mar 31;4(3):e4981. doi: 10.1371/journal.pone.0004981 (PMC2659687; doi:10.1371/journal.pone.0004981)
Supplement: Table S3 — The number of occurrences of domains in single(SD), multiple(MD) and as R (tandem repeats of domain) in a single or multiple chain are provided. R+MD includes domain occurrences that show repeating copies of self domain in multidomain contexts. (0.07 MB DOC) [file pone.0004981.s006.doc]

**Table S3 :** Domain contexts in rigid domain superfamilies. The numbers of occurrences of domains singly, multiply and as Repeats (tandem repeats of domain) in a single or multiple chain are provided. R+MD includes domain occurrences that show repeating copies of self domain in multidomain contexts. The average domain size (Av) and observed standard deviations in size (Dev) for every superfamily are also included

|  |  |  |  | Single chain | | | | Multiple chain | | | |  |
| --- | --- | --- | --- | --- | --- | --- | --- | --- | --- | --- | --- | --- |
| No | Superfamily | Av | Dev | Single Domain | Repeat (R ) | Multi Domain(MD) | R+  MD | Single Domain | Repeat | Multi Domain | R+ MD | Total Members |
| 1 | Cytochrome P450 | 417 | 31.4 | 11 | - | - | - | 10 | - | - | - | 21 |
| 2 | Terpenoid synthase | 323 | 24.6 | 1 | - | 1 | - | 5 | - | 1 | - | 8 |
| 3 | Nuclear receptor ligand binding domain | 250 | 14.8 | 10 | - | - | - | 14 | - | 1 | - | 25 |
| 4 | DNA glycosylase | 204 | 18.4 | 6 | - | 1 | - | 1 | - | 1 | - | 9 |
| 5 | Calponin homology domain, CH domain | 114 | 9.6 | 5 | 1 | - | - | - | 3 | - | - | 9 |
| 6 | TNF like | 145 | 7.0 | 1 | - | - | - | 5 | - | 1 | - | 7 |
| 7 | cAMP binding domain like | 135 | 3.2 | 1 | 2 | - | - | 2 | 1 | 3 | - | 9 |
| 8 | C2 domain | 133 | 7.4 | 5 | - | 2 | - | 1 | - | 2 | - | 10 |
| 9 | Actin crosslinking proteins | 118 | 5.0 | 1 | - | - | - | - | 1 | - | - | 2 |
| 10 | Invasin/Intimin cell adhesion fragments | 94 | 4.9 | - | - | - | 2 | - | - | - | - | 2 |
| 11 | Sm-like ribonucleoproteins | 75 | 4.6 | - | - | - | - | 6 | - | 1 | - | 7 |
| 12 | ALDH-like | 474 | 32.6 | 1 | - | - | - | 8 | - | - | - | 9 |
| 13 | Zn dependent exopeptidase | 299 | 18.6 | 5 | - | 10 | - | - | - | 5 | - | 20 |
| 14 | Purine and uridine phosphorylase | 254 | 20.1 | 1 | - | - | - | 8 | - | - | - | 9 |
| 15 | Metallo-hydrolase/oxidoreductase | 239 | 21.2 | 2 | - | - | - | 7 | - | 1 | - | 10 |
| 16 | Ribosome inactivating proteins | 253 | 8.2 | 4 | - | - | - | 4 | - | 3 | - | 11 |
| 17 | Lactate and malate dehydrogenase, C terminal domain | 167 | 8.0 | - | - | 7 | - | - | - | 9 | - | 16 |
| 18 | Superantigen toxins, C terminal domain | 111 | 5.1 | - | - | 1 | - | - | - | 7 | - | 8 |
| 19 | UBC like | 151 | 10.7 | 5 | - | - | - | 3 | - | 1 | - | 9 |
| 20 | DNA clamp | 124 | 6.6 |  | 1 | - | - | - | 6 | - | - | 7 |
| 21 | RNA binding domain, RBD | 87 | 7.4 | 9 | 6 | - | - | 4 | 1 | 1 | 1 | 22 |
| 22 | Metal binding domain | 70 | 2.5 | 6 | 1 | - | - | 2 | - | 1 | - | 10 |
| 23 | Interleukin 8 like chemokines | 70 | 8.9 | 8 | - | - | - | 13 | - | - | - | 21 |
| 24 | Chromo domain like | 67 | 5.2 | 3 | - | - | - | 4 | - | - | - | 7 |
|  | Total |  |  | 85 | 11 | 22 | 2 | 97 | 12 | 38 | 1 | 268 |
|  | % occurrence |  |  | 31.7 | 4.1 | 8.2 | 0.8 | 36.2 | 4.5 | 14.2 | 0.4 |  |
