# Supplementary material for: Length Variations amongst Protein Domain Superfamilies and Consequences on Structure and Function
Source: PLoS One. 2009 Mar 31;4(3):e4981. doi: 10.1371/journal.pone.0004981 (PMC2659687; doi:10.1371/journal.pone.0004981)
Supplement: Table S4 — Number of Protein-protein interactions (known and predicted) in length-deviant and length rigid domain superfamilies after searching in STRING databaseS30. (0.05 MB DOC) [file pone.0004981.s007.doc]

**Table S4**: Number of Protein-protein interactions (known and predicted) in

top-10 length-deviant and length rigid domain superfamilies after searching in STRING database^S30^

| **S.No** | **Domain superfamily** | **No of domain members with interaction information (in D/ Y/ O)** | **Total no of interactions (in D/Y/O) reported for each domain member** |
| --- | --- | --- | --- |
|  | **Length-deviant** |  |  |
| 1 | Phospholipase D nuclease | 2 | 28 |
| 2 | Lysozyme- like | 4 | 39 |
| 3 | Actin-like ATPase domain | 7 | 150 |
| 4 | RmlC-like cupins | 4 | 68 |
| 5 | Viral proteins | -- | -- |
| 6 | 6-phosphogluconate dehydrogenase C-terminal domain | 6 | 135 |
| 7 | *PRTase-like | 11 | 216 |
| 8 | *cytochrome-C | 22 | 221 |
| 9 | *S-adenosyl-L-methionine-dependent methyltransferases | 19 | 305 |
| 10 | Concanavalin A-like lectins/glucanases | 20 | 145 |
|  | **Total** | **95** | **1307** |
|  | **Length-rigid** |  |  |
| 1 | cAMP-binding domain-like | 4 | 49 |
| 2 | C2 domain Calcium/lipid-binding domain, CaLB | 10 | 167 |
| 3 | Cytochrome P450 | 8 | 149 |
| 4 | Terpenoid synthases | 4 | 41 |
| 5 | Nuclear receptor ligand-binding domain | 8 | 103 |
| 6 | DNA_ glycosylase | 5 | 104 |
| 7 | Calponin-homology domain, CH-domain | 5 | 94 |
| 8 | TNF-like | 6 | 57 |
| 9 | Actin-crosslinking proteins | 2 | 20 |
| 10 | Invasin/intimin cell-adhesion fragments | 2 | 16 |
|  | **Total** | **54** | **800** |

D,Y,O stand for interactions reported in the Drosophila, Yeast or closest homolog reported in the STRING database.

*: Highly interacting domains
